# iTRAQ-based quantitative proteomics analysis of the effect of ACT001 on non-alcoholic steatohepatitis in mice

**DOI:** 10.1038/s41598-023-38448-4

**Published:** 2023-07-13

**Authors:** Hui Zhou, Bin Niu, Xue Wu, Weike Chu, Yibing Zhou, Ze Chen, Yuqiang Mi, Yonggang Liu, Ping Li

**Affiliations:** 1 Clinical School of the Second People’s Hospital, Tianjin Medical University, Tianjin, China; 2 Department of Infectious Diseases, Xiangya Hospital, Central South University, Changsha, China; 3 Department of Infectious Diseases, First Hospital of Shanxi Medical University, Taiyuan, China; 4 Department of Hepatology, Tianjin Second People’s Hospital, Tianjin, China; 5 Tianjin Research Institute of Liver Diseases, Tianjin, China; 6 Department of Pathology, Tianjin Second People’s Hospital, Tianjin, China

**Keywords:** Non-alcoholic steatohepatitis, Drug discovery and development

## Abstract

ACT001 is a novel sesquiterpene lactone derivative that has been shown to have significant antitumor and anti-inflammatory effects. However, the effect of ACT001 on nonalcoholic steatohepatitis (NASH) is unknown. Methionine and choline deficient (MCD) diet induced NASH model in C57BL/6J mice. Steatosis, inflammation and fibrosis-related indices of serum and liver tissues were detected by fully automated biochemical analyzer, enzyme-linked immunosorbent assay (ELISA) kit, flow cytometry, hematoxylin and eosin (H&E), Masson and immunohistochemical staining. The results showed that ACT001 reduced serum lipid and inflammatory factor levels, attenuated hepatic steatosis, inflammation and fibrosis, and inhibited hepatic oxidative stress and activation of NOD-like receptor protein 3 (NLRP3) inflammatory vesicles in NASH mice. In addition, 381 differentially expressed proteins (DEPs), including 162 up-regulated and 219 down-regulated proteins, were identified in the MCD group and ACT001 high-dose group using isotope labeling relative and absolute quantification (iTRAQ) technique analysis. Among these DEPs, five proteins associated with NAFLD were selected for real-time fluorescence quantitative PCR (RT-qPCR) validation, and the results were consistent with proteomics. In conclusion, ACT001 has a therapeutic effect on NASH, and the results of proteomic analysis will provide new ideas for the mechanism study of ACT001 for NASH treatment.

## Introduction

Non-alcoholic fatty liver disease (NAFLD) is a spectrum of liver disease in which hepatic steatosis occurs in the absence of secondary causes of liver fat accumulation, such as drugs, heavy alcohol consumption, or certain genetic disorders^1^. Currently, the global prevalence of NAFLD is approximately 25%^2^. With the global epidemic of obesity and metabolic syndrome, the prevalence of NAFLD is increasing year by year and may become a major cause of end-stage liver disease worldwide in the future^3^. Non-alcoholic steatohepatitis (NASH), a progressive form of NAFLD, is characterized by the presence of more than 5% hepatic steatosis and inflammation with hepatocellular damage, with or without any fibrosis, which can progress to cirrhosis, end-stage liver disease and hepatocellular carcinoma^1,4^. In addition, NASH is associated with an increased risk of cardiovascular disease and type 2 diabetes^5^. Therefore, it is essential to develop effective therapeutic agents for NASH.

**Figure 1:**
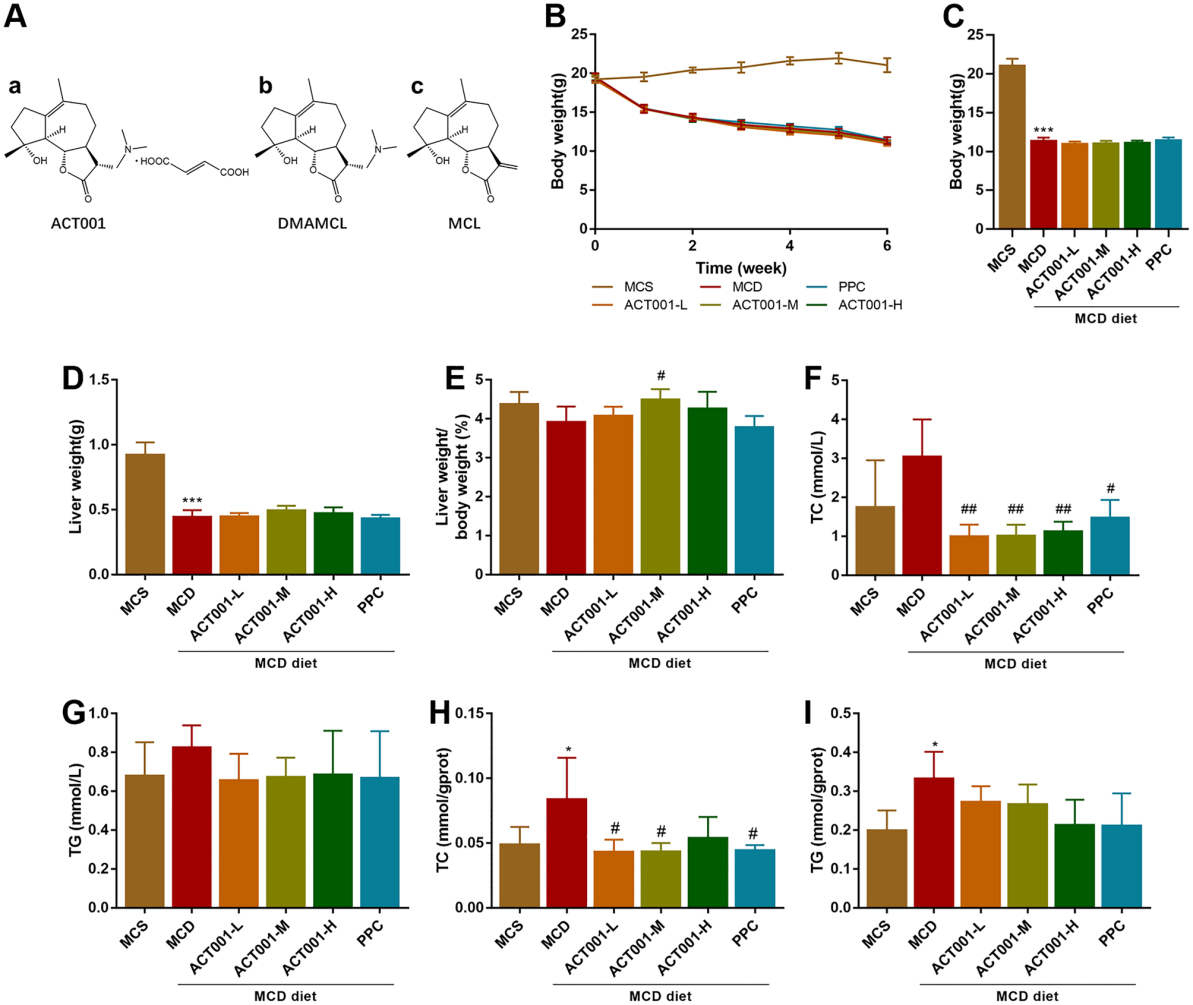
Effects of chemical structural formula of compounds and ACT001 on liver index and lipid levels in different groups of NASH mice. Chemical structure formulae (**A**) of ACT001 (**a**), DMAMCL (**b**) and MCL (**c**). Body weight changes after 2 weeks of MCD diet induction in C57BL/6 J mice and after continuing MCD diet and adding ACT001 or PPC treatment for 4 weeks (**B**). Body weight (**C**), liver weight (**D**), and liver weight/body weight percentage (**E**) in different groups of mice at the end of 6 weeks. Serum total cholesterol level (**F**), triglyceride level (**G**) in different groups of NASH mice. Total liver cholesterol levels (**H**), triglyceride levels (**I**) in different groups of NASH mice. MCS group, methionine choline sufficient diet group; MCD group, methionine and choline deficient diet group; ACT001-L, ACT001 low dose group; ACT001-M, ACT001 medium dose group; ACT001-H, ACT001 high dose group; PPC group, polyenyl phosphatidylcholine group. Compared with MCS group, ^*^p < 0.05, ^***^p < 0.001; compared with MCD group, ^#^*p* < 0.05, ^##^*p* < 0.01.

ACT001 is a novel sesquiterpene lactone derivative with anti-inflammatory and antitumor effects that has been designated by the US Food and Drug Administration (FDA) as an orphan drug for the treatment of glioma^6–9^. Dimethylaminomethyl lactone (DMAMCL) is a dimethylamino Michael adduct of Micheliolide (MCL), which releases MCL slowly in plasma and in vivo^10^. ACT001 is the fumarate form of DMAMCL with the molecular formula C17H27NO3-C4H4O4 (Fig. 1Aa). ACT001 is catalyzed by esterases to release MCL slowly and stably without significant toxicity to liver cells^10–12^, which provides a good basis for the future application of ACT001 in the treatment of liver lesions. In addition, studies have also confirmed the efficacy of ACT001 in other tumors (e.g., rhabdomyosarcoma^13^, leukemia^14,15^, breast cancer^16,17^, liver cancer^18^, etc.) and fibrosis (pulmonary fibrosis^19^, renal fibrosis^20^, peritoneal fibrosis^21^, etc.). However, the effect of ACT001 on NASH has not been reported. Polyenyl phosphatidylcholine (PPC), a harmless mixture containing a large number of unsaturated fatty acid groups derived from soybeans, may treat NASH through antioxidant, anti-inflammatory and anti-fibrotic effects^22,23^. In clinical practice, PPC can be used as an adjuvant therapy for the treatment of NAFLD and has a more significant improvement in fatty liver^24,25^. Therefore, we used PPC as a positive control drug for ACT001.

"Multiple parallel strikes" including insulin resistance, lipotoxicity, oxidative stress, endoplasmic reticulum stress and gut microbiota endotoxins are considered to be responsible for the progression of NASH, with oxidative stress considered to be a key factor in the progression of simple fatty liver to NASH^26,27^. In contrast, inflammatory response and concomitant fibrosis are considered to be key determinants of long-term prognosis in NASH^28^. Activation of NOD-like receptor protein 3 (NLRP3) inflammatory vesicles plays an important role in the development of inflammation and fibrosis in NASH^29^. Interestingly, NLRP3 inflammatory vesicle activation transforms hepatic stellate cells (HSCs) into an activated pro-fibrotic state^30^. Extracellular signals from resident cells (e.g., macrophages, hepatocytes, hepatic sinusoidal endothelial cells) and inflammatory cells can further modulate HSCs activation to promote fibrosis development^31^. Therefore, chemicals with antioxidant, anti-inflammatory and anti-fibrotic effects may hold promise for the prevention or treatment of NASH.

Proteomic analysis can be used to study and identify proteins in tissues under therapeutic conditions, to identify possible biomarkers of disease, to detect potential therapeutic targets, and to understand the underlying biological functions^32^. Isobaric tagging proteomics techniques for relative and absolute quantification (iTRAQ) have been widely used in the study of NAFLD^33–35^. Therefore, we employed iTRAQ technology to elucidate the mechanism of action of ACT001 for the treatment of NASH.

Here, we explored the effects of different doses of ACT001 on methionine- and choline-deficient (MCD) diet-induced NASH mice. The iTRAQ-based quantitative proteomic analysis revealed the differential protein expression of ACT001 for NASH treatment, and analyzed the main functions and possible pathways of action of the differential proteins, which laid the foundation for the selection of subsequent drug therapeutic targets and the study of molecular mechanisms.

## Results

### Effect of ACT001 on blood lipid levels and liver lipid content in MCD-induced NASH mice

As expected, MCD induced a decrease in body weight, liver weight and liver weight/body weight percentage in NASH mice (Fig. 1B–E), and the body weight and liver weight of NASH mice did not return to normal after 4 weeks of ACT001 treatment, while the liver weight/body weight percentage increased in the ACT001-M group, with statistical differences (Fig. 1E). The results indicated that ACT001 inhibited the decrease of liver organ coefficients induced by MCD feed in mice.

Mice in the MCD group exhibited elevated serum total cholesterol (TC) and triglyceride (TG) levels (Fig. 1F,G). Compared with the MCD group, the ACT001 (L, M, H) group and the PPC group reduced serum TC levels, which was statistically different (Fig. 1F). In addition, the ACT001 (L, M, H) group and the PPC group also reduced serum TG levels, but there was no statistical difference (Fig. 1G). The results indicated that MCD diet caused an increase in serum TC and TG levels in mice, while ACT001 reduced TC and TG levels, and the differences between the low, medium and high dose groups were not significant. In addition, we also examined the contents of TC and TG in the liver tissues of NASH mice. Compared with the MCS group, the liver TC and TG contents of mice in the MCD group increased with statistical differences; compared with the MCD group, the liver TC contents of mice in the ACT001-L, ACT001-M and PPC groups decreased with statistical differences, and the decrease of TC contents in the ACT001-H group was not obvious (Fig. 1H). Compared with the MCD group, the liver TG content of mice in the ACT001 (L, M, H) group and the PPC group decreased, but there was no statistically significant difference (Fig. 1I).

### Effect of ACT001 on liver steatosis in MCD-induced NASH mice

**Figure 2:**
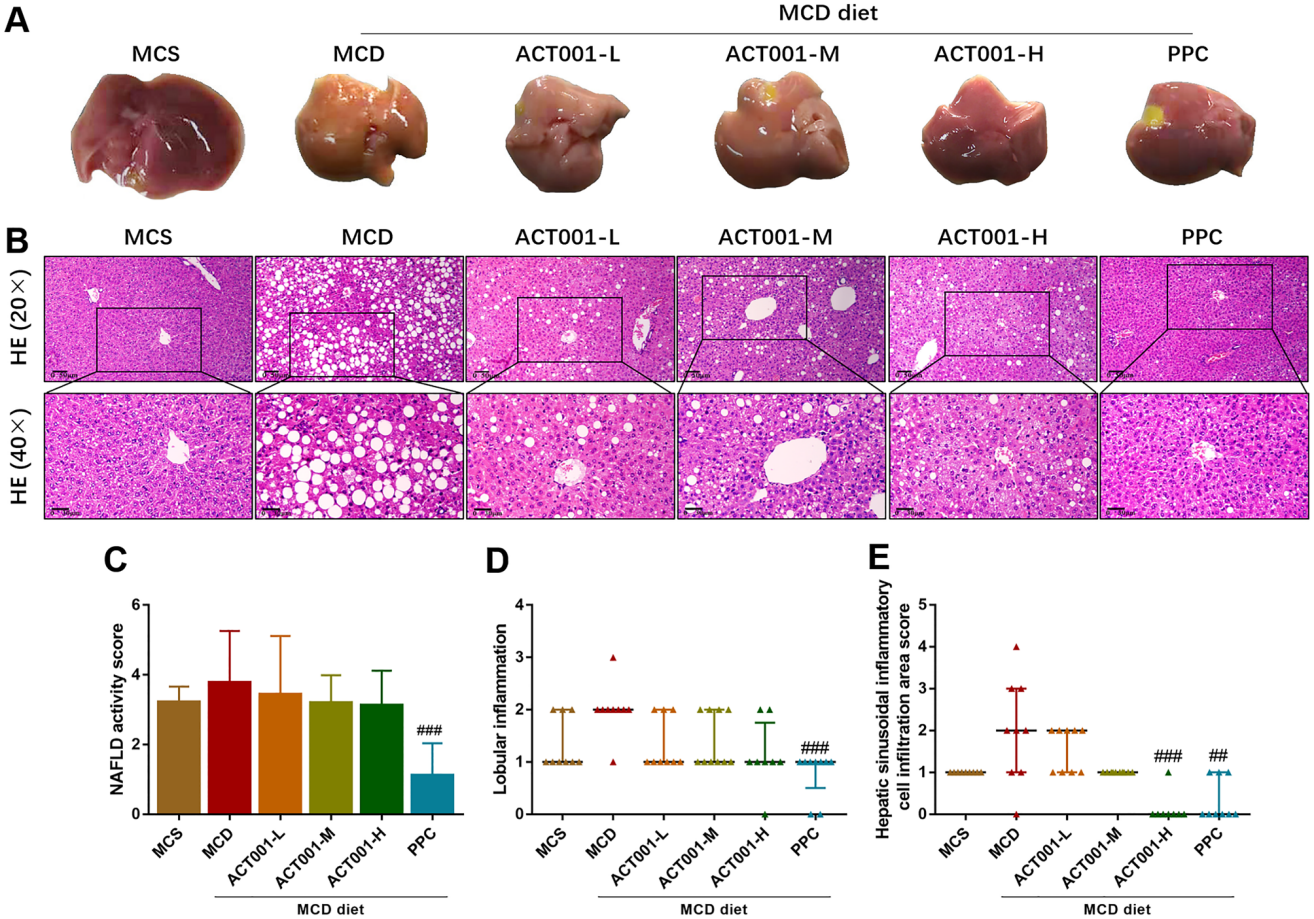
ACT001 reduces hepatic steatosis in MCD-induced NASH mice. Gross liver morphology of different groups of NASH mice (**A**), representative images of H&E staining (**B**), NAFLD activity score (**C**), inflammation score in NAFLD activity score (**D**), and inflammatory cell infiltration score of liver sinusoids (**E**). MCS group, methionine choline sufficient diet group; MCD group, methionine and choline deficient diet group; ACT001-L, ACT001 low dose group; ACT001-M, ACT001 medium dose group; ACT001-H, ACT001 high dose group; PPC group, polyenyl phosphatidylcholine group. 20× with a scale of 50 μm; 40× with a scale of 30 μm. Compared with the MCD group, ^##^*p* < 0.01, ^###^*p* < 0.001.

The liver of mice in the MCS group was dark red in color, with smooth surface and sharp edges, while the liver of mice in the MCD group was reduced in size, light yellow in color, with greasy surface and blunt edges compared with the MCS group. The livers of mice in ACT001 (L, M, H) and PPC groups showed little difference in volume compared with the MCD group, but the color gradually approached dark red, with smooth surface and sharp edges (Fig. 2A).

The results of H&E staining showed that the liver tissues of mice in the MCD group exhibited significant macrovesicular steatosis compared with the MCS group. The size and number of lipid droplets in the liver tissues of mice in the ACT001 (L, M, H) and PPC groups were reduced compared with those in the MCD group (Fig. 2B). The results of NAFLD activity score showed that mice in the ACT001 (L, M, H) group were reduced compared with those in the MCD group, while those in the PPC group were significantly and statistically different (Fig. 2C). The results suggest that ACT001 improves liver steatosis in NASH mice.

### ACT001 attenuates MCD diet-induced inflammation and NLRP3 inflammatory vesicle activation in NASH mice

In the H&E staining results, inflammation scores were increased in the MCD group compared to the MCS group; inflammation scores were decreased in the ACT001 (L, M, H) and PPC groups compared to the MCD group, with a statistical difference in the PPC group (Fig. 2D). Since we observed during the reading of liver sections that the inflammation score in the NAFLD activity score did not reflect well the inflammatory effect of the MCD model (Fig. 2D), and the infiltration of hepatic sinusoidal inflammatory cells was more pronounced in the liver sections of mice fed the MCD diet and significantly improved by ACT001 or PPC treatment, especially in the ACT001-H and PPC groups (Fig. 2E). Therefore, we scored the area of hepatic sinusoidal inflammatory cell infiltration and generated our scoring criteria.

**Figure 3:**
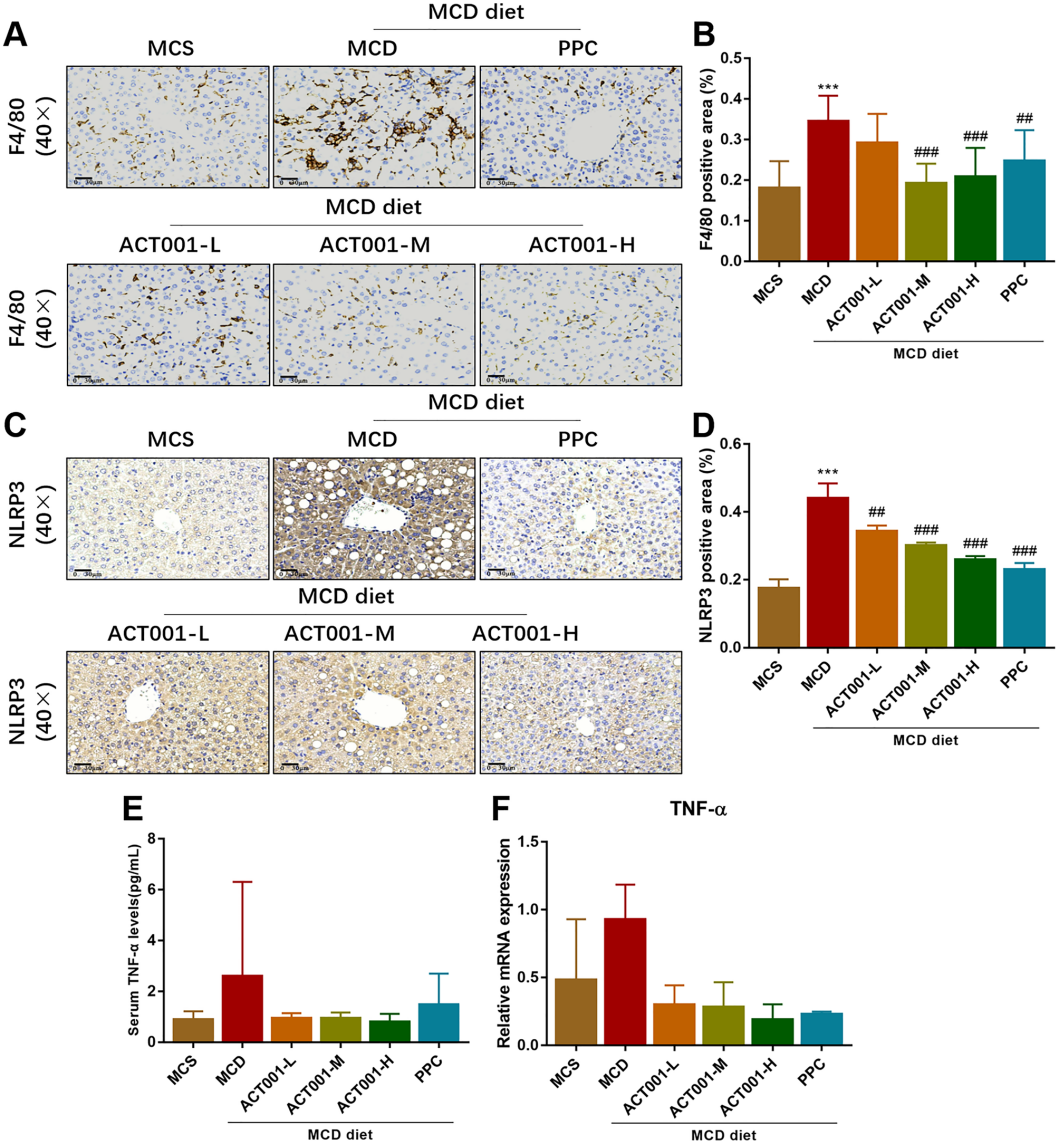
ACT001 attenuates MCD diet-induced inflammation and activation of NLRP3 inflammatory vesicles in NASH mice. Immunohistochemical staining of F4/80 cells in liver sections of different groups of NASH mice (**A**), quantitative analysis of positive regions (**B**). Immunohistochemical staining to observe the expression of NLRP3 (**C**) and quantitative analysis of positive regions (**D**). Flow cytometric analysis of serum TNF-α levels in different groups of NASH mice (**E**). Hepatic TNF-α mRNA levels in different groups of NASH mice (**F**). MCS group, methionine choline sufficient diet group; MCD group, methionine and choline deficient diet group; ACT001-L, ACT001 low dose group; ACT001-M, ACT001 medium dose group; ACT001-H, ACT001 high dose group; PPC group, polyenyl phosphatidylcholine group. 40× with a scale of 30 μm. Compared with MCS group, ^***^p < 0.001; compared with MCD group, ^#^*p* < 0.05, ^##^*p* < 0.01, ^###^*p* < 0.001.

Aggregation of hepatic macrophages (including local Kupffer cells and macrophages derived from circulating monocytes) and overproduction of pro-inflammatory cytokines are important pathological features of NASH^36^. In the present experiment, we observed that the expression of immunohistochemical staining for the hepatic macrophage infiltration marker F4/80 was significantly upregulated in the MCD group of mice, whereas F4/80 expression was significantly downregulated in the ACT001-M, ACT001-H and PPC groups, with statistical differences (Fig. 3A,C).

NLRP3 inflammatory vesicles play an important role in the pathogenesis and progression mechanism of NASH^37^. We examined NLRP3 protein expression in mouse liver tissues using immunohistochemical staining. NLRP3 protein expression was significantly increased in liver tissues of mice in the MCD group compared to the MCS group. Compared with the MCD group, the NLRP3 protein expression in the liver tissues of mice in the ACT001 (L, M, H) and PPC groups was decreased with statistical differences, and the groups showed a dose-dependent pattern (Fig. 3B,D).

In addition, by flow cytometry, we observed that serum inflammatory factor TNF-α levels were upregulated in the MCD group mice compared to the MCS group. Serum TNF-α levels were reduced in mice in the ACT001 (L, M, H) group compared to the MCD group (Fig. 3E). In addition, the levels of hepatic TNF-α mRNA were elevated in the MCD group mice compared with the MCS group, and ACT001 treatment inhibited the expression of IL-6 and TNF-α mRNA in a dose-dependent manner (Fig. 3F). Our results showed that feeding MCD diets induced liver inflammation in mice, while ACT001 treatment significantly attenuated liver inflammation and reduced the levels of inflammatory cytokines, significantly in the ACT001-M and ACT001-H groups.

### ACT001 inhibits oxidative stress and lipid peroxidation in NASH liver

**Figure 4:**
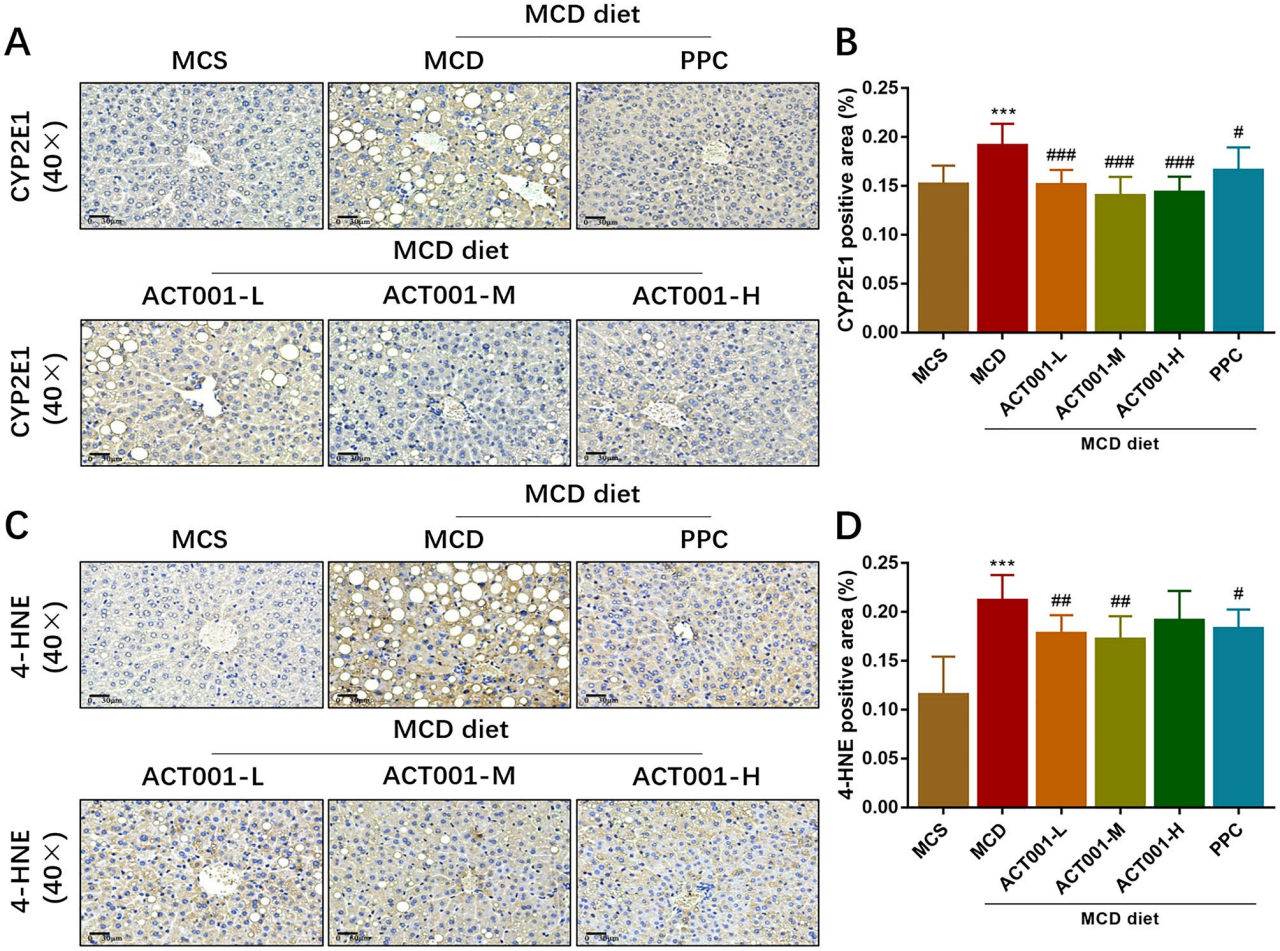
ACT001 inhibits oxidative stress and lipid peroxidation in NASH liver. Immunohistochemical staining and quantitative analysis of CYP2E1 in liver sections from different groups of NASH mice (**A**, **B**); Immunohistochemical staining of 4-HNE in liver sections from different groups of NASH mice (**C**, **D**). MCS group, methionine choline sufficient diet group; MCD group, methionine and choline deficient diet group; ACT001-L, ACT001 low dose group; ACT001-M, ACT001 medium dose group; ACT001-H, ACT001 high dose group; PPC group, polyenyl phosphatidylcholine group. 40 × with a scale of 30 μm. Compared with the MCS group, ^***^p < 0.001; compared with the MCD group, ^###^*p* < 0.001.

It is well known that cytochrome P450 2E1 (CYP2E1) plays a key role in the generation of ROS^38^. 4-hydroxy-2-nonenal (4-HNE) is a lipid peroxidation product and is often used as an indicator to determine lipid peroxidation. To observe the changes in the expression of CYP2E1 and 4-HNE in MCD diet-induced NASH mice after ACT001 treatment, we performed immunohistochemical staining of liver sections. Compared with the MCS group, the area of positive area of CYP2E1 staining in liver sections of mice in the MCD group was significantly increased, whereas the area of positive area in liver sections of mice in the ACT001 (L, M, H) and PPC groups was significantly decreased (Fig. 4A,B). Similarly, the area of positive area of 4-HNE staining in liver sections of mice in the MCD group was significantly increased, while the area of positive area in liver sections of mice in the ACT001 (L, M, H) and PPC groups was significantly decreased (Fig. 4C,D). Our results suggest that ACT001 inhibits hepatic oxidative stress and lipid peroxidation in NASH mice in a dose-dependent manner.

### ACT001 ameliorates MCD diet-induced NASH-associated fibrosis

**Figure 5:**
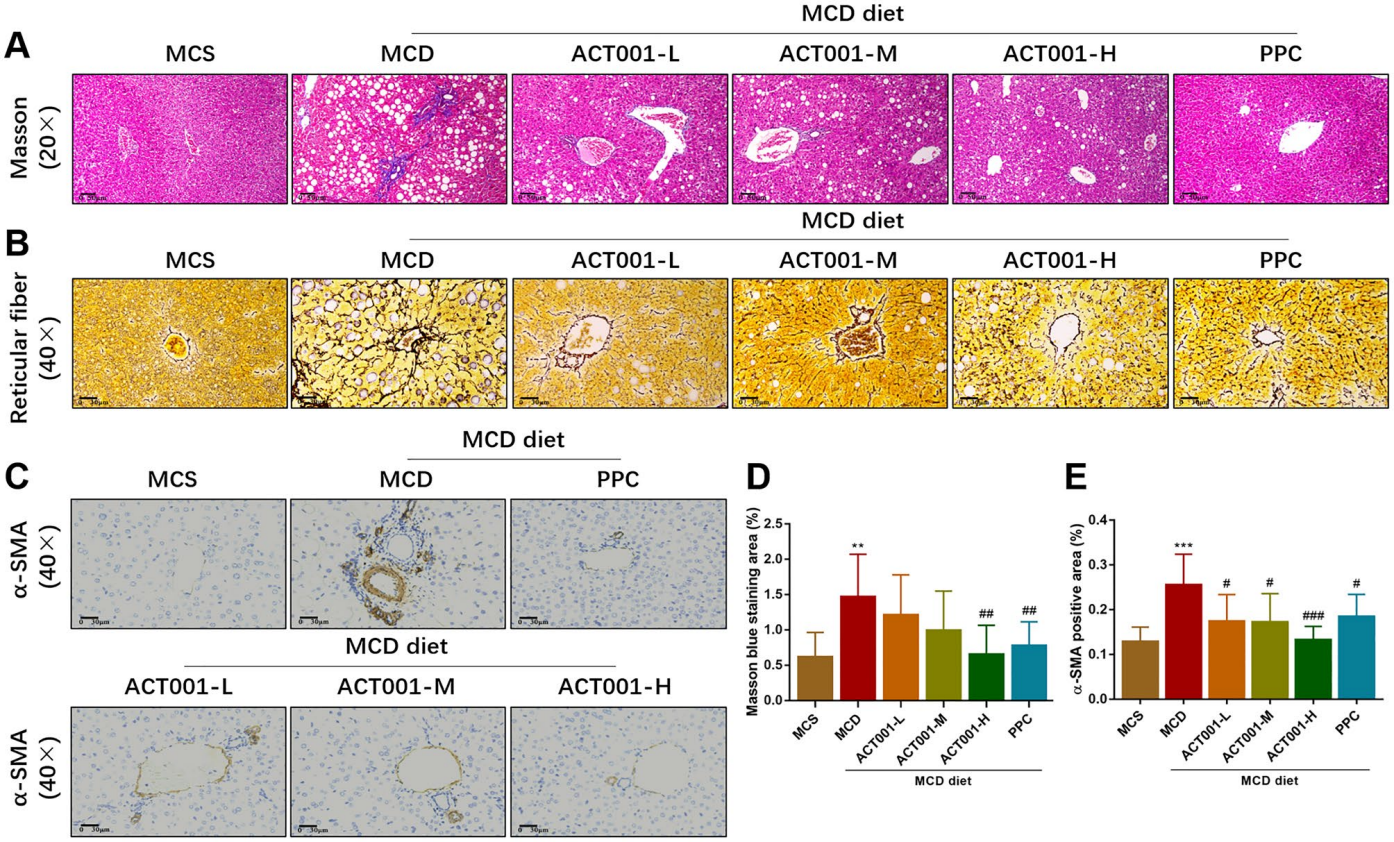
ACT001 ameliorates MCD diet-induced NASH-associated fibrosis. Representative images of Masson trichrome staining of liver sections from different groups of NASH mice (20×) (**A**). Representative images of reticulocyte staining of liver sections from different groups of NASH mice (40×) (**B**). Representative images of α-SMA immunohistochemical staining of liver sections from different groups of NASH mice (40×) (**C**). Quantification of collagen fiber staining (blue stained area) (20×) (**D**). Quantification of the area of positive stained area for α-SMA immunohistochemical staining (40×) (**E**). MCS group, methionine choline sufficient diet group; MCD group, methionine and choline deficient diet group; ACT001-L, ACT001 low dose group; ACT001-M, ACT001 medium dose group; ACT001-H, ACT001 high dose group; PPC group, polyenyl phosphatidylcholine group. 20× with a scale of 50 μm; 40× with a scale of 30 μm. Compared with the MCS group, ^**^p < 0.01; compared with the MCD group, ^#^*p* < 0.05, ^##^*p* < 0.01, ^###^*p* < 0.001.

To investigate the effect of ACT001 on MCD diet-induced fibrosis in mice, histological analysis was performed using Masson trichrome staining, reticulocyte staining and α-SMA immunohistochemical staining.Masson trichrome staining and quantitative analysis showed that there was significant collagen deposition in the MCD group compared to the MCS group. Compared with the MCD group, the ACT001 (L, M, H) and PPC groups had reduced collagen deposition, with statistically significant differences between the ACT001-H and PPC groups (Fig. 5A,D). Reticulofibrillar staining showed slight fibrous hyperplasia in the confluent area of the MCD group, while ACT001 treatment reduced fibrous hyperplasia (Fig. 5B). α-SMA immunohistochemical staining showed that the area of positive area was significantly higher in the MCD group than in the MCS group, i.e., the activity of HSCs was significantly increased. In contrast, the ACT001 (L, M, H) group and the PPC group had significantly decreased active HSCs and were statistically different, most significantly in the ACT001-M, ACT001-H and PPC groups (Fig. 5C,E). Our results suggest that ACT001 reduces hepatic collagen deposition and hepatic stellate cell activation in NASH mice induced by MCD feed.

### Differential expression of proteins

**Figure 6:**
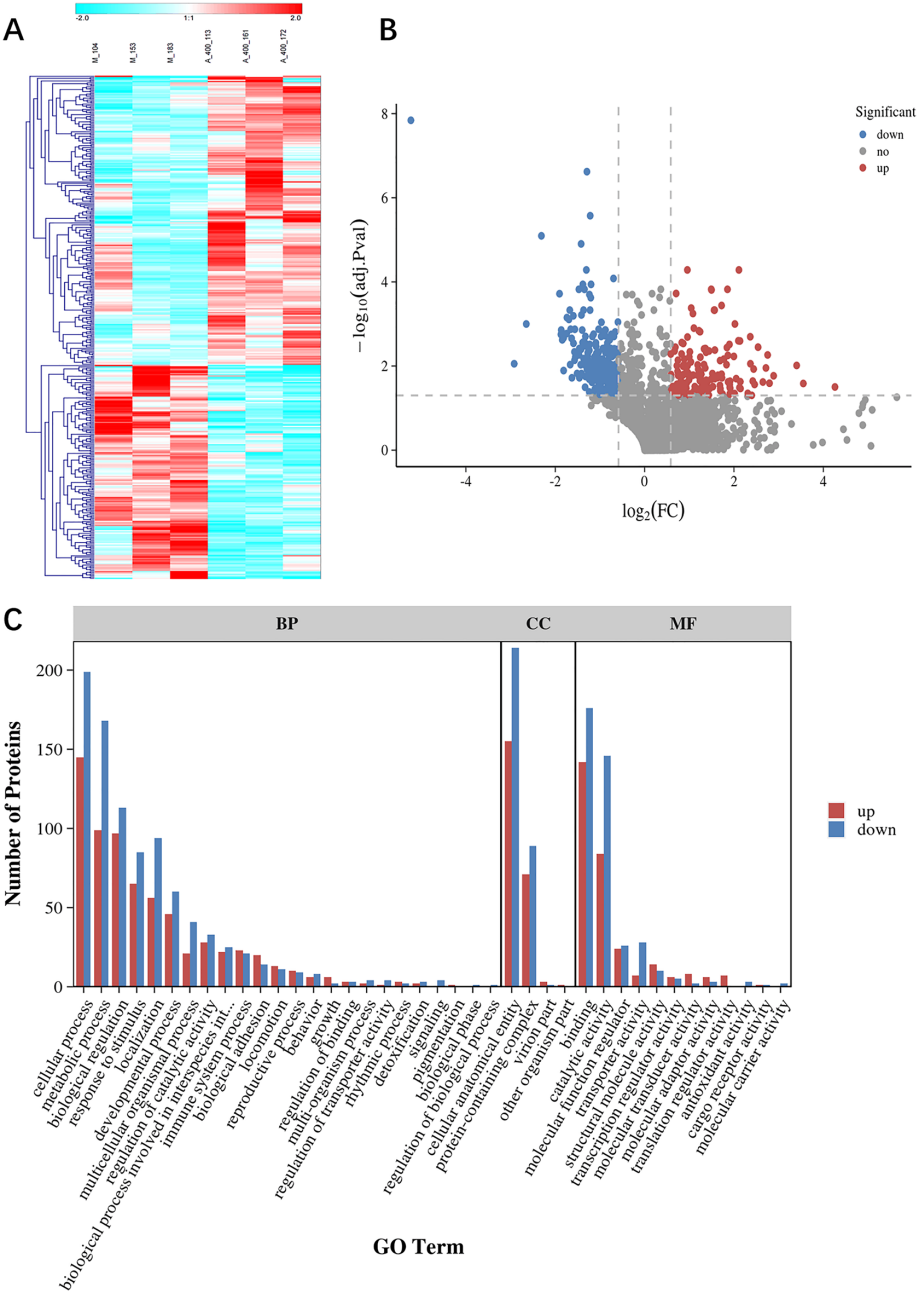
Histogram of differential protein expression and GO secondary classification annotations. hierarchical clustering analysis of DEPs between MCD group and ACT001-H (**A**). Red represents up-regulated expression and blue represents down-regulated expression. 113 (M-104), 114 (M-153) and 115 (M-183) are the three biological replicates of the MCD group. 116 (A-400-113), 117 (A-400-161) and 118 (A-400-172) are the three biological replicates of the ACT001-H group. mcd group versus the ACT001-H group in the volcano plot (**B**). Significantly up- or down-regulated DEPs are indicated in red or blue, respectively, and gray indicates proteins with no statistical difference. The bars depict the 3 components of GO analysis, including biological process (BP), cellular component (CC) and molecular function (MF) (**C**). The vertical coordinate indicates the number of proteins, and red or blue indicates the number of up- or down-regulated proteins, respectively.

The results of cluster analysis showed significant differences between the MCD and ACT001-H groups, indicating that protein levels differed between the two groups (Fig. 6A). 3097 proteins with unique sequences were identified in the MCD and ACT001-H groups. Compared with the ACT001-H group, 381 differentially expressed proteins were identified in the MCD group, including 162 up-regulated proteins and 219 down-regulated proteins (Supplementary Table S4). Red represents proteins that were significantly differentially up-regulated (FC ≥ 1.5, *p* < 0.05), blue represents proteins that were significantly down-regulated (FC ≤ 0.67, p < 0.05), and gray represents proteins with no difference or no statistical significance (Fig. 6B).

### GO and KEGG enrichment analysis of differentially expressed proteins

**Figure 7:**
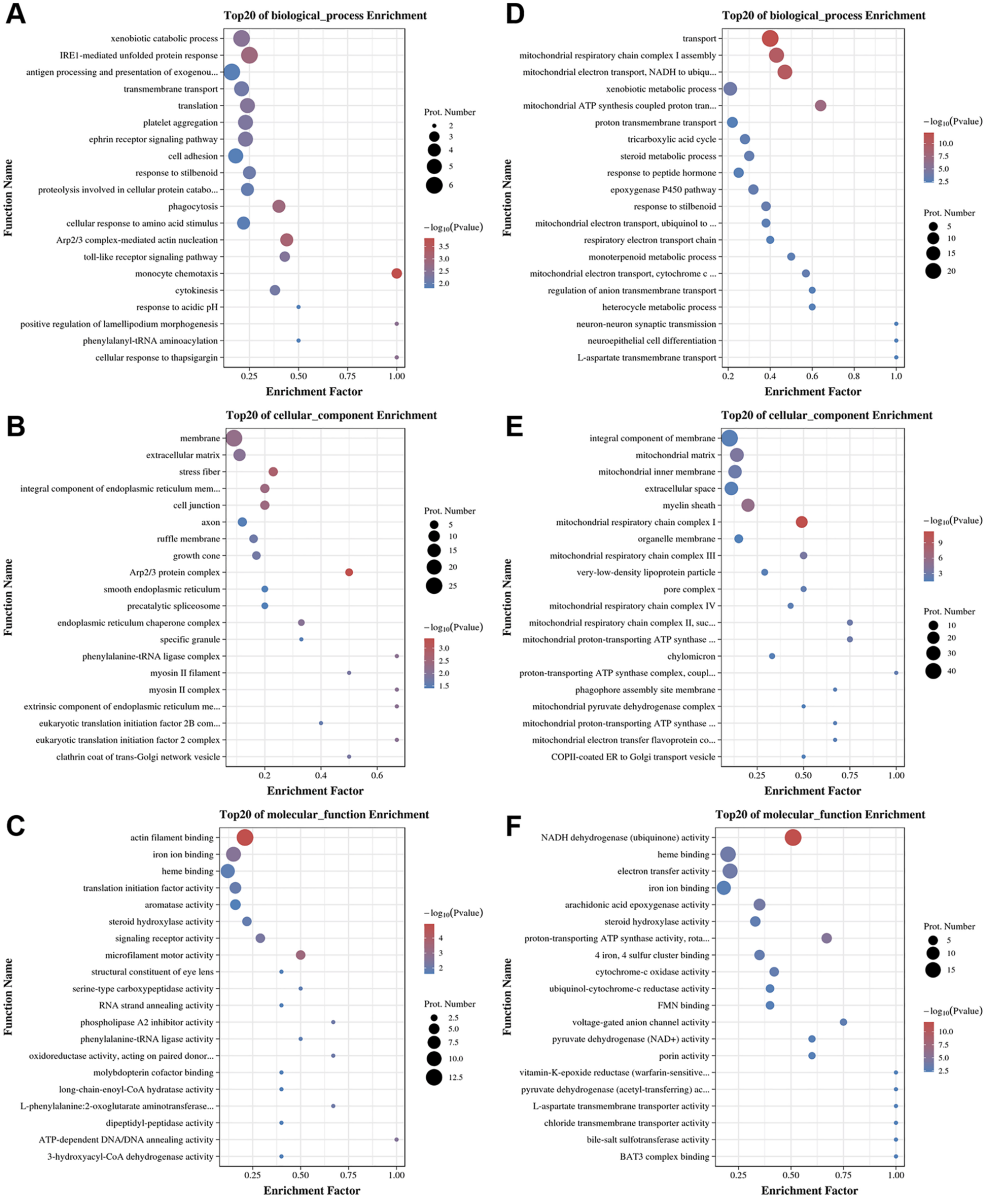
GO analysis of differentially expressed proteins. Bubble plots of GO enrichment BP analysis for up-regulated DEPs (**A**), CC analysis bubble plots (**B**), and MF analysis bubble plots (**C**). Down-regulated GO enrichment BP analysis bubble plots of DEPs (**D**), CC analysis bubble plots (**E**), MF analysis bubble plots (**F**). The larger the bubble is, the more protein there is. The color of bubbles indicates the *p*-value, the smaller the *p*-value, the greater the significance.

The GO classification analysis of DEPs was performed and the up-regulated and down-regulated functional classifications were compared (Fig. 6C). The results showed that the up- and down-regulation of DEPs involved a total of 40 functions in the GO functional classification. The DEPs were mainly focused on cellular processes, metabolic processes, biological regulation, cellular anatomical entities, protein-containing complexes, binding and catalytic activities. We examined the hypergeometric distribution of the BP, CC and MF parts of the up-regulated and down-regulated DEPs, respectively, and then presented the classification of the top 20 up-regulated DEPs and the top 10 down-regulated DEPs in bubble plots, respectively. We found that the GO enrichment analysis terms of up-regulated DEPs were mainly monocyte chemotaxis, Arp2/3 protein complex and actin filament binding (Fig. 7A–C). The GO enrichment analysis terms for down-regulated DEPs were mainly transport, mitochondrial respiratory chain complex I assembly, mitochondrial electron transport chain, mitochondrial respiratory chain complex I, and NADH dehydrogenase (ubiquitin) activity (Fig. 7D–F).

**Figure 8:**
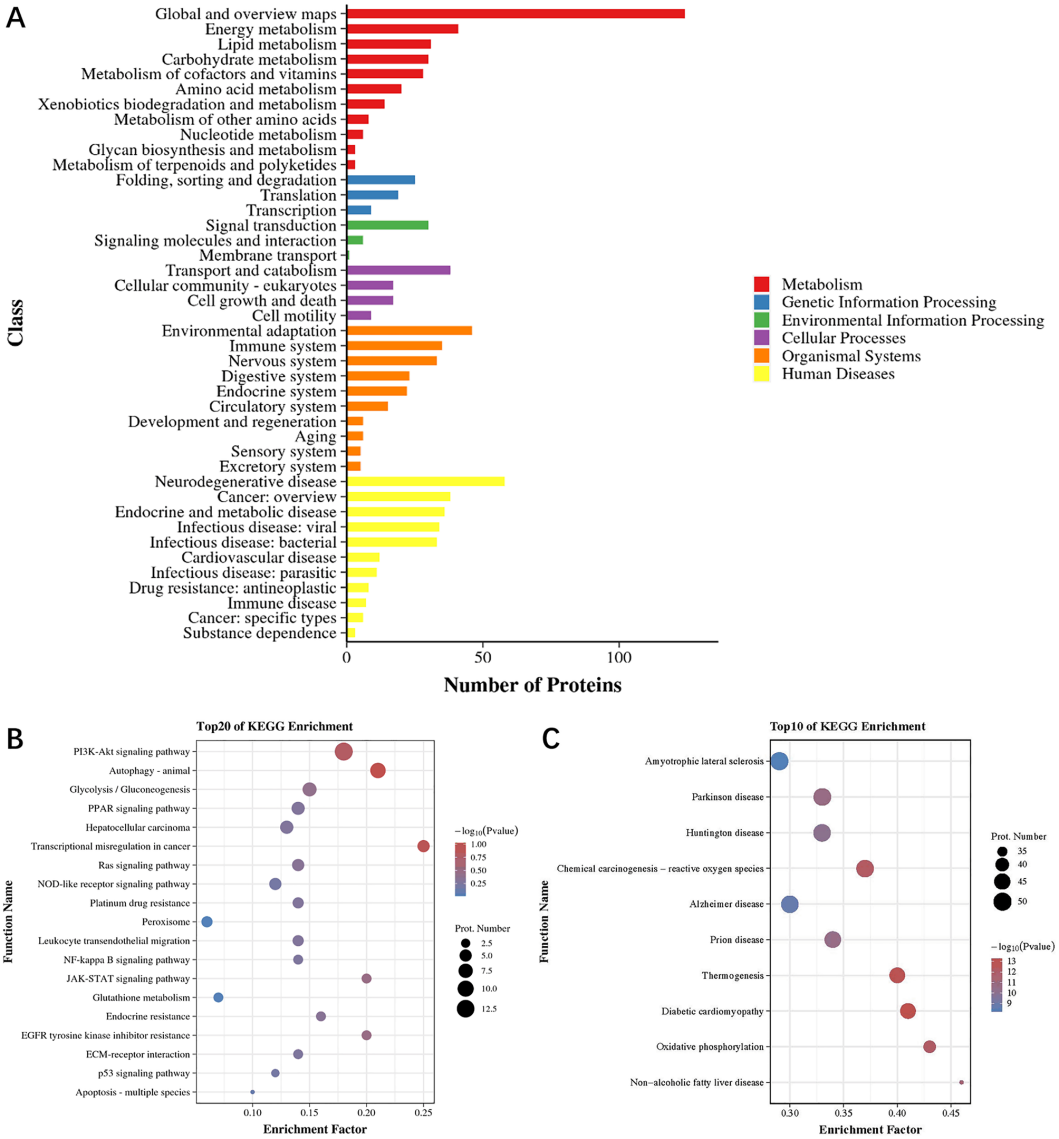
KEGG analysis of differentially expressed proteins. Annotated histogram of KEGG classification (**A**). The horizontal coordinate is the number of proteins and the vertical coordinate is the annotated classification. Bubble plot of Pathway enrichment analysis for the top 20 of the upregulated DEPs (**B**). Plot of the top 10 Pathway enrichment analysis bubbles in the down-regulated DEPs (**C**). The larger the bubble, the more protein there is. The color of the bubbles indicates the *p*-value, the smaller the *p*-value, the greater the significance.

According to the annotated distribution of KEGG secondary classification, we found that DEPs were mainly concentrated in global and overview maps, energy metabolism, lipid metabolism, transport and catabolism, environmental adaptation, immune system, nervous system, neurodegenerative diseases, cancer, endocrine and metabolic diseases, and infectious diseases (viral or bacterial) (Fig. 8A). The top 20 up-regulated and top 10 down-regulated DEPs in the MCD group compared to the ACT001-H group are listed in Supplementary Table S3, and we performed KEGG enrichment pathway analysis on these DEPs, which showed that the up-regulated DEPs are mainly involved in transcriptional dysregulation, autophagy, PI3K-Akt signaling pathway (Fig. 8B), and down-regulated DEPs were mainly involved in processes such as non-alcoholic fatty liver disease, oxidative phosphorylation, diabetic cardiomyopathy, and thermogenesis (Fig. 8C).

### Validation of differentially expressed proteins

**Figure 9:**
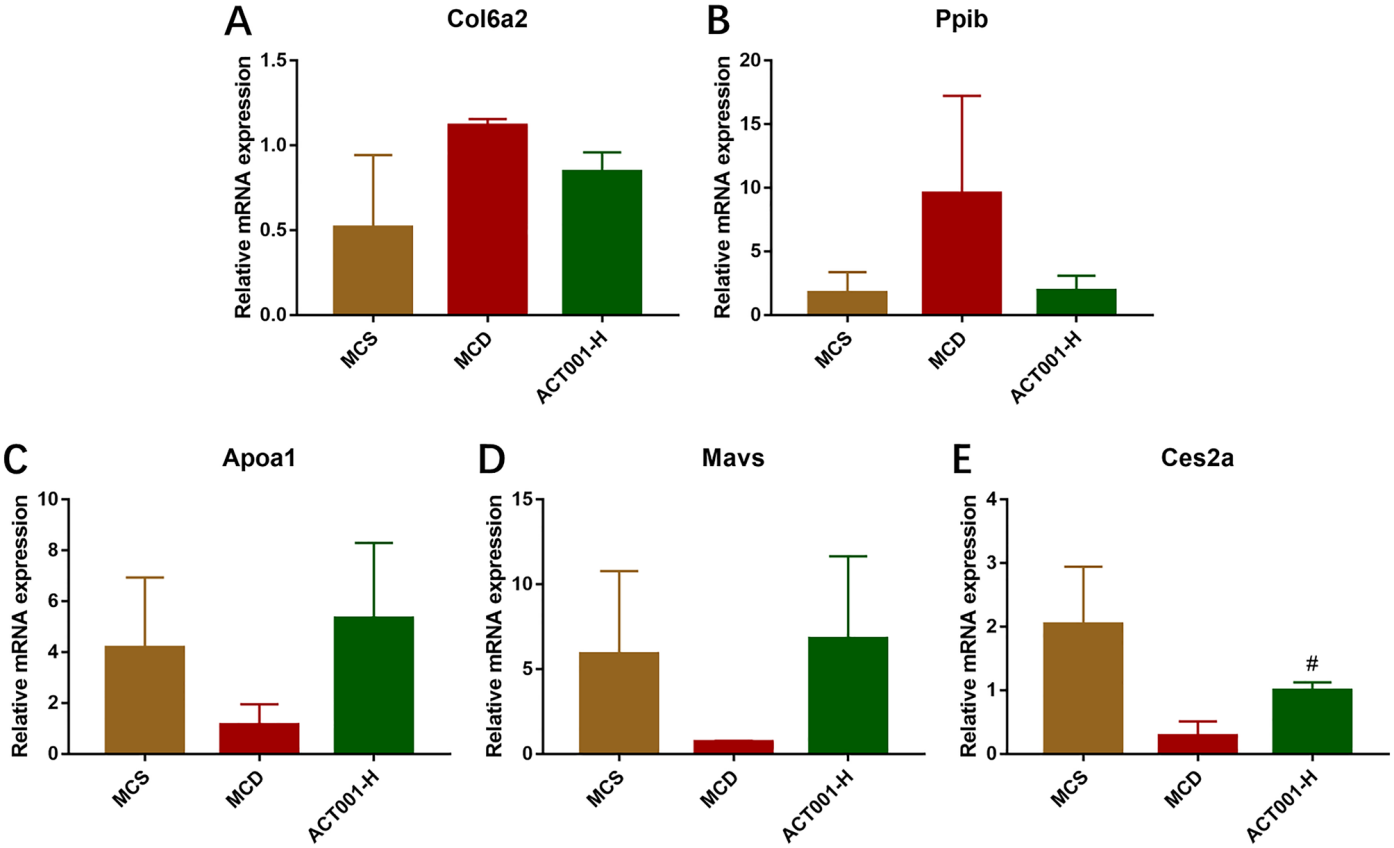
Validation of differentially expressed proteins. Hepatic Col6α2 (**A**), Ppib (**B**), Apoa1 (**C**), Mavs (**D**) and Ces2a (**E**) mRNA expression levels in different groups of NASH mice. MCS group, methionine choline sufficient diet group; MCD group, methionine and choline deficient diet group; ACT001-H, ACT001 high dose group. Compared with the MCD group, ^#^*p* < 0.05.

To confirm the identified differential proteins, we randomly selected 2 proteins from the first 20 up-regulated DEPs and 3 proteins from the first 10 and last 10 down-regulated DEPs for validation, respectively, for a total of 5 DEPs: including collagen type VI α2 chain (COL6A2), peptidyl prolyl isomerase B (PPIB), apolipoprotein A1 (APOA1), mitochondrial antiviral signaling protein (MAVS), and carboxylesterase 2A (CES2A). In the next experiments, we performed protein validation at the transcriptional level in the MCS, MCD and ACT001-H groups of mice. The results showed that the expression levels of hepatic Col6α2 and Ppib mRNA were elevated in the MCD group mice compared with the MCS group, while the expression levels of hepatic Col6α2 and Ppib mRNA were decreased in the ACT001-H group mice compared with the MCD group (Fig. 9A,B). The hepatic Apoa1, Mavs and Ces2a mRNA expression levels were decreased in the MCD group compared with the MCS group. The expression levels of Apoa1, Mavs and Ces2a mRNA were increased in the ACT001-H group compared with the MCD group (Fig. 9C–E), with statistically significant upregulation of Ces2a mRNA expression in the ACT001-H group compared with the MCD group (Fig. 9E).

## Discussion

Currently, in addition to diet control and increased exercise, some NASH patients require pharmacological treatment; however, there is no approved pharmacological treatment. PPC is a polyunsaturated phospholipid extract derived from soybeans that has antioxidant properties and is more commonly used as an adjunct to the clinical treatment of toxic liver disease; however, it is not approved by the FDA for the treatment of NASH and has some side effects, with occasional gastrointestinal disturbances at high doses, such as complaints of stomach upset, soft stools and diarrhea, and in rare cases, allergic reactions. In this study, we investigated the therapeutic effects and possible mechanisms of action of ACT001 in NASH mice. Our results showed that ACT001 improved hepatic steatosis, inflammation and fibrosis in MCD diet-induced NASH and inhibited hepatic oxidative stress, activation of NLRP3 inflammatory vesicles and activation of HSCs in the ACT001-M and ACT001-H groups. However, the underlying molecular mechanism of ACT001 amelioration of NASH is unclear, for which we used iTRAQ quantitative proteomics to study the changes of proteins before and after ACT001 treatment. The results showed that the differential proteins before and after ACT001 treatment for NASH were closely associated mainly with transcriptional dysregulation, autophagy, PI3K-Akt signaling pathway, JAK-STAT signaling pathway, EGFR tyrosine kinase inhibitor activity, non-alcoholic fatty liver disease, oxidative phosphorylation, diabetic cardiomyopathy, thermogenesis, and chemical carcinogenesis-reactive oxygen species in cancer.

Hepatic lipid accumulation is due to lipid uptake or ab initio lipid synthesis over fatty acid oxidation or lipoprotein secretion^39^. In the present study, we found that ACT001 reduced the number of hepatic macrovesicular steatosis in NASH mice and also reduced serum TC, TG and Glu levels, with lower TC and Glu levels in the ACT001 (L, M, H) group than in the MCS group, which may be related to the MCD diet. Because previous studies have shown that ACT001 dose-dependently reverses the age-related decline in Glu-God and reduces TC levels^11^, we speculate that the malnutrition status induced by the MCD diet may synergistically lower total cholesterol levels and counteract the reversal of blood glucose by ACT001 to the extent that TC and Glu levels are lower than in normal controls, a situation may change with the dietary transformations used to construct the NASH model. In addition, we demonstrated that ACT001 inhibited the expression of lipid synthesis gene Srebp-1c and its target gene Fas mRNA, promoted the expression of fatty acid β-oxidation genes Pparα and Cpt1α mRNA, improved hepatic steatosis and reduced lipid levels. The positive control drug PPC also showed a good effect in improving steatosis by a mechanism similar to that of ACT001. PPC inhibited the expression of lipid synthesis genes Srebp-1c and Fas mRNA and increased the expression of fatty acid β-oxidation gene Cpt1α mRNA.

Oxidative stress is a state in which oxidative effects exceed antioxidant effects in vivo. Oxidative stress can promote the progression of hepatic inflammation in NASH by stimulating adaptive immune responses, and targeting mitochondrial oxidative stress may be a good candidate for NASH treatment^27,40^. Hepatic CYP2E1 is increased in NASH patients compared to normal liver^41^. It has been demonstrated that NASH can be improved by decreasing CYP2E1 activity and expression and inducing antioxidant activity to alleviate oxidative stress^42–44^. In addition, hepatic expression of 4-HNE is considered a reliable marker of lipid peroxidation in NAFLD^45,46^. Our results showed that ACT001 inhibited the elevated hepatic CYP2E1 and 4-HNE expression in MCD diet-induced NASH mice with antioxidant effects. Some studies have demonstrated that PPC can reduce ROS content in NASH rats^22^. In the present experiment, we found that PPC could also downregulate the expression of CYP2E1 and 4-HNE, thus exerting antioxidant effects. In conclusion, ACT001 can improve NASH by inhibiting oxidative stress.

The occurrence of MCD diet-induced inflammation in NASH mice is associated with the activation of macrophages, in addition to oxidative stress^47^. Macrophage activation releases various inflammatory cytokines, such as IL-6 and TNF-α^48^. The identification of hepatic macrophages is based on the expression of F4/80^49^. Depletion of hepatic macrophages ameliorates steatohepatitis and inhibits liver inflammation and fibrosis^50^. Immunohistochemical staining showed that both ACT001 and PPC resulted in a significant reduction in the number of F4/80 + infiltrating macrophages in the liver in NASH, suggesting a strong anti-inflammatory effect of ACT001. It has been shown that blocking NLRP3 inflammatory vesicle activation reduces hepatic inflammation and fibrosis in experimental NASH^29^. In addition, ACT001 exerts an anti-neuritis effect by inhibiting NLRP3 inflammatory vesicle activation^9^. Our study also confirmed that ACT001 inhibits the activation of NLRP3 inflammatory vesicles, thereby suppressing liver inflammation in NASH.

Activation of HSCs from various causes has been identified as a central driver of fibrosis in NASH^31^. In the normal liver, HSCs do not express α-SMA when they are in a resting state, and α-SMA activity and expression increase after HSCs activation^51^. A previous study reported that MCL can improve renal fibrosis by inhibiting the MTDH/BMP/MAPK pathway^20^. Our study also demonstrated that ACT001 reduced collagen deposition and expression of pro-fibrotic growth factors in liver tissue of NASH mice, suggesting that ACT001 ameliorates NASH-associated fibrosis by inhibiting HSCs activation.

Collagen type VI alpha 2 chain (COL6A2) is one of the three alpha chains of COL6 (COL6α1, COL6α2, and COL6α3)^52^. It has been shown that Zac1 binding to TGF-β and COL6A2 promoter is directly involved in the pathophysiological mechanisms of NAFLD, driving the formation of fibrosis in vivo^53^. In addition, it has also been demonstrated that all-COL6 pro fibers can control adipocyte function and are closely associated with obesity-related metabolic diseases^54^. Our experimental results also showed that ACT001 inhibited MCD-induced hepatic Col6α2 mRNA expression in NASH, which is consistent with previous findings. Peptidyl prolyl isomerase B (PPIB) is a member of the peptidyl prolyl cis–trans isomerase (PPIase) family of molecules^55^. It has been demonstrated that PPIB plays an important role in the response to inflammatory stimuli and oxidative stress, and it can be involved in tissue or systemic inflammation^56^. Liu L et al. also found that PPIB independently induces macrophage scorching and leads to NLRP3 inflammatory vesicle activation^57^. In addition, PPIB and the pro-apoptotic factor CHOP are upregulated by oxidative stress in gastric cancer cells, activating the JAK2/STAT3 signaling pathway and inhibiting miR-520d-5p expression, while PPIB further enhances the JAK2/STAT3 signaling pathway, thus promoting gastric cancer cell proliferation^58^. Our proteomic results showed that PPIB was expressed in the NASH model significantly expressed in liver, and ACT001 suppressed PPIB expression, which we also validated at the transcriptional level. We speculate that the inflammatory suppressive effect of ACT001 may also be mediated through PPIB, which needs to be verified by more experiments in the future.

Apolipoprotein A1 (ApoA1), encoded by the APOA1 gene, is a major component of plasma high-density lipoprotein (HDL)^59^. Previous studies have shown that foods high in carotenoids such as spinach and tomatoes can increase APOA1 expression and its increased expression can reduce hepatic cholesterol content, thereby ameliorating high-fat diet-induced hepatic steatosis in rats^60^. The proteomic validation results in this study suggest that ACT001 may improve NASH by promoting hepatic APOA1 expression, which requires extensive future experimental validation. The MAVS gene encodes an essential intermediate protein in the viral-triggered β-interferon signaling pathway, which is required for the activation of transcription factors that regulate β-interferon expression and contribute to antiviral innate immunity^61^. It has recently been shown that MAVS in hepatocytes plays a role in the prevention of NAFLD by helping to regulate healthy mitochondrial function, and this study suggests that targeting MAVS may be a new avenue for the treatment of NAFLD^62^. Our proteomic analysis is also consistent with the results that MAVS expression is downregulated in the liver of NASH mice and MAVS expression is upregulated after ACT001 intervention, suggesting that ACT001 may ameliorate NASH by targeting MAVS.CES2A is a member of the subfamily of mammalian carboxylesterases (CES) that catalyze the hydrolysis of esters, amides, thioesters, and carbamates^63^. In addition, CES2 has been shown to be a potent diglyceride and monoglyceride lipase, which is reduced in expression in obese human and mouse livers, and knocking it down leads to hepatic steatosis^64^. In our present study, the expression of CES2A was reduced in the liver of NASH mice and was upregulated after ACT001 treatment, suggesting that ACT001 may promote the expression of CES2A and thus improve hepatic steatosis. The results of the PCR assay were consistent with those obtained by proteomics, indicating the reliability of proteomic analysis, and the results also suggest that ACT001 may improve hepatic steatosis by affecting certain target proteins (e. g. APOA1, MAVS and CES2A) to improve NASH.

The present study has some limitations. The MCD diet model used in the current experiment can produce the most severe NASH phenotype, i.e., hepatic macrovesicular steatosis, inflammation, and fibrosis in a relatively short period of time^65^, while MCD diet mice show significant weight loss and no insulin resistance, which is different from what is commonly referred to as NASH^66^. We considered choosing a diet model closer to human NASH in future studies. Biological variability within and between individuals of NASH mice in proteomic analysis may bias the proteomic results and affect the reproducibility of the measurements. In fact, we selected multiple samples from different groups for testing during the study, which in principle avoided the occurrence of large errors and ensured the reliability of the proteomics measurements. If future reproducibility studies are conducted, the sample size can be further expanded to verify the reliability of the proteomics results of this study. In addition, we selected only the MCD and ACT001 high-dose groups, and did not analyze the MCS and PPC groups. Because the focus of this study was mainly to analyze the therapeutic effect and potential targets of ACT001 on MCD diet-induced NASH mice, we focused more on the differential protein comparison between the MCD group and ACT001 high-dose group when performing the proteomic analysis. In addition, the volcano plot was not log-normally distributed in the results of proteomics analysis, which may be a result of the small sample size, but from the objective results, the protein expressions of the MCD group and ACT001-H group were statistically significant, so we hope to conduct replicate experiments with large sample size in future studies to verify the reliability of the results of proteomics analysis.

## Conclusion

In this study, the therapeutic effects of different doses of ACT001 on a mouse model of NASH were investigated for the first time. Low, medium and high doses of ACT001 were beneficial for the treatment of NASH, with medium and high doses of ACT001 being preferred. In improving inflammation, oxidative stress and liver fibrosis, ACT001 showed a significant dose-dependent effect. In contrast, ACT001 treatment groups did not show a significant dose dependence in improving liver steatosis and lipids, but it is easy to see that ACT001 treatment was effective in improving liver steatosis and reducing lipids. In addition, our study comprehensively analyzed the protein expression patterns and biological process changes involved in the improvement of NASH by ACT001 through quantitative proteomics. It is particularly interesting that five proteins, COL6A2, PPIB, APOA1, MAVS and CES2A, may become new targets of ACT001 for the treatment of NASH, and the signaling pathways involved need to be further validated in subsequent experiments to provide clues and basis for the study of the molecular mechanism of ACT001 for the treatment of NASH. In conclusion, ACT001 is expected to be a new drug candidate for the treatment of NASH, with potential clinical applications and important scientific implications. In the future, we will carry out further validation experiments of the target proteins and conduct transcriptomic analysis to clarify the specific target pathways of the target proteins.

## Materials and methods

### Drugs and diet

ACT001 was generously provided by Shangde Pharmaceutical Technology Co., LTD (Tianjin, China), lot numbers C10668-10 and C10668-11. Polyenyl phosphatidylcholine (PPC) was purchased from Sanofi Pharmaceutical Co., LTD (Beijing, China), lot no. ABJD465B. Both ACT001 and PPC were dissolved in 0.9% saline before use. MCD and methionine and choline sufficient (MCS) feeds were purchased from HuafuKang Biotechnology Co.,LTD (Beijing, China). Each 1000 g of MCS feed contains 171.4 g of amino acid premix (without methionine), 3 g of methionine, 2 g of choline, 455.58 g of sucrose, 150 g of corn starch, 50 g of maltodextrin, 30 g of cellulose, 100 g of corn oil, 35 g of mineral salt mixture M1001, 3 g of calcium bicarbonate, 5 g of vitamin mixture V1003, and antioxidant TBHQ 0.02 g. Each 1000 g of MCD feed contains 171.4 g of amino acid premix (without methionine), 455.58 g of sucrose, 150 g of corn starch, 50 g of maltodextrin, 30 g of cellulose, 100 g of corn oil, 35 g of mineral salt mixture M1001, 3 g of calcium bicarbonate, 5 g of vitamin mixture V1003, and 0.02 g of antioxidant TBHQ.

### Animals and experimental design

Healthy 8-week-old C57BL/6J mice (20 ± 2 g) were purchased from HuafuKang Biotechnology Co.,LTD (Beijing, China). Acclimatization feeding was performed for 1 week before the start of the experiment. The animals were housed in the Animal Center of the Institute of Radiological Medicine, Chinese Academy of Medical Sciences, at a controlled temperature of 22 ± 2 °C, relative humidity of 55–65%, alternating light and dark cycles of 12 h, and free access to water and food.

At the beginning of the experiment, mice were randomly divided into two groups: the MCS diet group (MCS) (n = 10) and the model group (n = 52). The MCS group was given MCS diet and the model group was given MCD diet for 2 weeks each. At the end of week 2, we cervically dislocated and executed two model group mice and removed the livers for H&E staining. At 20× magnification, we observed significant hepatic steatosis, focal necrosis and balloon-like degeneration in both model group mice (Supplementary Fig. S1). At this point, we considered that the NASH model had been successfully established. Next, we divided the model group into five subgroups (n = 10/group): (1) MCD diet group (MCD): mice were fed MCD diet and saline; (2) ACT001 low dose group (ACT001-L): mice were fed MCD diet and 100 mg kg^−1^ d^−1^ ACT001; (3) ACT001 medium dose group (ACT001-M): mice fed MCD diet and 200 mg kg^−1^ d^−1^ ACT001; (4) ACT001 high dose group (ACT001-H): mice fed MCD diet and 400 mg  kg^−1^ d^−1^ ACT001; (5) Polyenyl phosphatidylcholine (PPC) group: mice fed MCD diet and 150 mg  kg^−1^ d^−1^ PPC. All groups stopped administration at the end of the 6th week. All treatments were administered by gavage and the doses were calculated according to the body weight of the mice. The technical route is recorded in Supplementary Figure S2.

Body weights were recorded daily until the end of the experiment. Mice were executed by cervical dislocation after 12 h fasting on the last day, and blood was collected from the eye veins, centrifuged at 3000 g for 15 min at 4 ℃ and serum was retained for serum biochemical and cytokine assays. The livers were collected and weighed, and some liver tissues were cut and fixed in freshly prepared 10% formalin for histological analysis. The remaining liver tissue was rapidly frozen in liquid nitrogen and stored at − 80 °C for molecular biology analysis. The liver index is the ratio of liver weight to body weight.

### Serum biochemical and cytokine analysis

Serum total cholesterol (TC) and triglyceride (TG) levels were measured by a Tokyo Hitachi fully automated biochemical analyzer. The content of TC and TG in liver tissue homogenates was determined according to the instructions of the enzyme-linked immunosorbent assay (ELISA) kit (Nanjing Jiancheng). Serum tumor necrosis factor-α (TNF-α) levels were measured by flow cytometry (BD, USA).

### Histological analysis

Liver tissues were fixed in 10% formalin, embedded in paraffin, and sectioned 5 μm thick for hematoxylin–eosin (H&E) staining, Masson trichrome staining, and reticulocyte fiber staining, respectively. According to the NAFLD activity score, histological variables were scored on a scale ranging from 0 to 8 (steatosis 0–3, intralobular inflammation 0–3, hepatocellular swelling 0–2), 0–2 for simple fatty liver, 3–4 for probable NASH, and > 5 for definite NASH^67,68^. In addition, we scored the hepatic sinusoidal inflammatory cell infiltration according to the observed hepatic sinusoidal inflammatory cell infiltration and Kupffer cell hyperplasia as follows: 0, rare hepatic sinusoidal inflammatory cell infiltration and Kupffer cell hyperplasia; 1, slightly increased hepatic sinusoidal inflammatory cell infiltration and Kupffer cell hyperplasia with a small focal distribution (< 25% hepatic sinusoidal area); 2, increased hepatic sinusoidal inflammatory cell infiltration and Kupffer cell hyperplasia with a focal distribution (25% to 50% hepatic sinusoidal area); 3 points, both hepatic sinusitis cell infiltration and Kupffer cell hyperplasia were more obvious and showed multifocal distribution (> 50% hepatic sinusoidal area); 4 points, both hepatic sinusitis cell infiltration and Kupffer cell hyperplasia were very obvious and showed diffuse distribution (> 75% hepatic sinusoidal area).

Masson trichrome stained sections were analyzed using Image J V1.8.0 software (National Institutes of Health) to quantify the degree of fibrosis. Each group included at least three liver sections with five randomly selected high magnification (20×) fields of view for each section. Stained sections were independently evaluated by a liver histopathologist experienced in the histological assessment of NAFLD who had no knowledge of the source of the sections.

### Immunohistochemistry

After dewaxing, liver sections were subjected to antigen repair, serum blocking, incubation with anti-F4/80 (1:500, CST, USA), α-smooth muscle actin (α-SMA) (1:6000, Abcam, UK), NLRP3 (1:1000, Abcam, UK), cytochrome P450 2E1 (CYP2E1) (1:200. Abcam, UK) and 4-hydroxynonenal (4-HNE) (1:100, Abcam, UK) primary antibodies and biotin-labeled secondary antibodies were incubated serially, followed by the addition of a chromogenic agent to detect positive staining, and all sections were restained with hematoxylin. Sections were scanned with a fully automated digital scanning system (PRECICE 500, China), and brown staining was considered positive for antibodies. Image J V1.8.0 software was used to quantify the positive areas. Each group consisted of at least three liver sections, with five randomly selected high magnification (40×) fields of view per section. The process of taking images was performed by an assessor blinded to the treatment assignment. Antibody information are shown in Supplementary Table 1.

### Real-time quantitative PCR

Total RNA was extracted from mouse liver using RNA Extraction Solution (Servicebio, WuHan) and reverse transcribed into cDNA using a reverse transcription kit (Servicebio, WuHan) as a template. the reaction system was configured according to the instructions for use and PCR amplification was performed. 95 °C 30 s pre-denaturation, cycling parameters 95 °C 15 s and 60 °C 30 s were cycled 40 times. Relative expression was calculated using the 2-ΔΔCt method with GAPDH as a control. Primers for relevant genes are shown in Supplementary Table 2.

### Protein extraction and quantification

The liver tissue was well ground and transferred to a centrifuge tube, and the supernatant was extracted by adding an appropriate amount of lysis buffer (phosphate buffer solution containing a final concentration of 1% phenylmethylsulfonyl fluoride) and centrifuged at 12,000 g for 10 min after sonication. Add an equal volume of acetonitrile (ACN) solution, precipitate and centrifuge the supernatant, freeze and dry until the volume is reduced by half, then centrifuge at 10,000 g for 20 min using a pre-wetted 10 kD ultrafiltration tube (Merck Millipore, UFC501096, Germany), then repeat the above operation with 200 µL of ultrapure water, collect the penetration solution and adjust the pH. Take 2 μL of protein sample, dilute to an appropriate multiple, and determine the protein concentration by Bradford method, as follows: configure 2 mL of 0.02 μg/μL BSA standard. BSA standards were taken 0 μL, 20 μL, 40 μL, 60 μL, 80 μL, 100 μL, 120 μL, 140 μL, 160 μL, 180 μL, 200 μL into a new 1.5 mL EP tube, add the diluent to 200 µL, mix well. Take 20 μL of diluted BSA solution into the microplate, and set up 3 replicate wells for each concentration. Add 20 μL of the diluted protein sample to the microplate, and set up 3 replicate wells for each sample. Add 180 μL of G250 color developing solution to each well, and develop at room temperature in the dark for 5 min. Microplate reader was used to determine the absorbance value of A580. Calculate the concentration of the protein sample based on the standard curve and the dilution factor of the protein sample.

### Protein digestion

The protein is dissolved and diluted, centrifuged by shaking, digested with trypsin (trypsin: protein = 1:100) and incubated at 37 °C. 1 mL of methanol activates the C18 column material, centrifuged by shaking and the supernatant is discarded. Acidify, centrifuge and discard supernatant. Peptide samples were acidified, shaken and vortexed and added to centrifuge tubes, mixed silently for 30 min, centrifuged and supernatant discarded. Then the samples were washed twice with 0.1% Formic Acid (FA) + 3% ACN for desalting and eluted with 1 mL of 0.1% FA + 80% ACN. The eluted peptides were dried with a vacuum concentrator.

### iTRAQ labeling and fractionation

Dissolve the peptide sample and shake and centrifuge. Labeling was performed according to the iTRAQ-8 labeling kit (SCIEX) instructions, with labels 113, 114 and 115 for the MCD group and labels 116, 117 and 118 for the ACT001-H group. After the samples were labeled and mixed, the mixed peptides were then separated by applying the Ultimate 3000 HPLC system (Thermo DINOEX, USA) for graded separation of the peptide samples. The chromatographic column used was a Welch C18 column (250 × 4.6 mm 5 mm). The peptides were separated using a gradual increase in ACN concentration under alkaline conditions, combined into 12 fractions, and the combined fractions were desalted on a Strata-X column and dried under vacuum.

### Liquid chromatography-tandem mass spectrometry (LC–MS/MS) analysis

Peptide samples on the machine. The mass spectrometry data acquisition was performed using a Triple TOF 5600 + liquid mass spectrometry system (AB SCIEX, USA). Peptide samples were dissolved in 2% acetonitrile/0.1% formic acid and analyzed using a Triple TOF 5600 plus mass spectrometer coupled to an Eksigent nanoLC system (AB SCIEX, USA). The peptide solution was added to a C18 capture column (3 µm, 350 µm × 0.5 mm, AB Sciex, USA) and gradient eluted on a C18 analytical column (3 µm, 75 µm × 150 mm, Welch Materials, Inc) with a 90 min time gradient at a flow rate of 300 nL/min. For IDA (information-dependent acquisition), primary mass spectra were scanned with an ion accumulation time of 250 ms, and secondary mass spectra of 30 precursor ions were acquired with an ion accumulation time of 50 ms. MS1 spectra were acquired in the range of 350–1200 m/z, and MS2 spectra were acquired in the range of 100–1500 m/z. The dynamic exclusion time of precursor ions was set to 15 s.

### Database search and bioinformatics analysis

The mass spectrometry downstream data were searched for databases using ProteinPilot and analyzed for iTraq quantification. The database used in this case was the UniProt database (http://www.uniprot.org/). DEPs were identified using a fold change (FC) threshold, with *p* values < 0.05 considered significant after adjustment, FC ≥ 1.5 for expression upregulation and FC ≤ 0.67 for expression downregulation. GO annotations (http://www.geneontology.org/)^69–71^ were performed on selected DEPs to classify them functionally according to three major terms: biological process, cellular component and molecular function, and Fisher's exact test was applied to identify GO entries that were significantly enriched for DEPs. KEGG annotation (http://www.genome.jp/kegg/) was performed to identify the most significant biochemical metabolic pathways and signal transduction pathways in which DEPs are involved^72–74^. All bioinformatics images were drawn by R language software. R version 4.0.2. June 2020. The R Project for Statistical Computing. https://www.r-project.org/. 22/06/2020.

### Statistical analysis

Statistical analysis was performed using SPSS 26.0 software, and GraphPad Prism 7 software was used for graphing. Data obeying normal distribution were expressed as mean ± standard deviation (SD); one-way analysis of variance (ANOVA) was used for comparison between multiple groups, followed by Bonferroni or Tamhane T2 multiple post hoc comparisons. Skewed data were expressed as median ± interquartile interval and Kruskal–Wallis H was used for between-group comparisons, *p* < 0.05 were considered statistically significant. In iTRAQ quantitative proteomic analysis, proteins with *p* < 0.05 and difference folds (FC) ≥ 1.5 or ≤ 0.67 were considered DEPs. GO and KEGG analyses were performed using Fisher's exact test, and categories and pathways with *p* < 0.05 were considered statistically significant.

### Ethical statement

This animal protocol was approved by the Animal Ethics Committee of Nankai University (Approval No. 2021-SYDWLL-000003). The study was carried out in compliance with the ARRIVE guidelines. All methods were carried out in accordance with relevant guidelines and regulations.

## Supplementary Information

Supplementary Information 1.

Supplementary Table S4.

## Data Availability

The datasets generated and/or analysed during the current study are available in the [PRIDE] repository, [http://www.ebi.ac.uk/pride/archive]. Dataset identifier PXD035890 (Reviewer account details: username: reviewer_pxd035890@ebi.ac.uk, password: xRt1Jpdk).

## References

[1] Chalasani N (2018). The diagnosis and management of nonalcoholic fatty liver disease: Practice guidance from the American Association for the Study of Liver Diseases. Hepatology.

[2] Younossi ZM (2016). Global epidemiology of nonalcoholic fatty liver disease-Meta-analytic assessment of prevalence, incidence, and outcomes. Hepatology.

[3] Younossi Z (2018). Global burden of NAFLD and NASH: Trends, predictions, risk factors and prevention. Nat. Rev. Gastroenterol. Hepatol..

[4] Chalasani N (2012). The diagnosis and management of non-alcoholic fatty liver disease: Practice Guideline by the American Association for the Study of Liver Diseases, American College of Gastroenterology, and the American Gastroenterological Association. Hepatology.

[5] Harvey BE (2022). NASH: Regulatory considerations for clinical drug development and U.S. FDA approval. Acta Pharmacol. Sin..

[6] Guo J (2019). Dimethylaminomicheliolide (DMAMCL) suppresses the proliferation of glioblastoma cells via targeting pyruvate kinase 2 (PKM2) and rewiring aerobic glycolysis. Front. Oncol..

[7] Li Q (2020). ACT001 modulates the NF-κB/MnSOD/ROS axis by targeting IKKβ to inhibit glioblastoma cell growth. J. Mol. Med. (Berl.).

[8] Tong L (2020). ACT001 reduces the expression of PD-L1 by inhibiting the phosphorylation of STAT3 in glioblastoma. Theranostics.

[9] Liu Q (2020). Anti-neuroinflammatory effects of dimethylaminomylide (DMAMCL, i.e., ACT001) are associated with attenuating the NLRP3 inflammasome in MPTP-induced Parkinson disease in mice. Behav. Brain Res..

[10] Zhang Q (2012). Guaianolide sesquiterpene lactones, a source to discover agents that selectively inhibit acute myelogenous leukemia stem and progenitor cells. J. Med. Chem..

[11] Sun Z (2019). Long-term every-other-day administration of DMAMCL has little effect on aging and age-associated physiological decline in mice. Aging (Albany NY).

[12] Xi X-N (2019). Pharmacokinetics, tissue distribution and excretion of ACT001 in Sprague-Dawley rats and metabolism of ACT001. J. Chromatogr. B Anal. Technol. Biomed. Life Sci..

[13] Xu N (2019). The anti-tumor growth effect of a novel agent DMAMCL in rhabdomyosarcoma in vitro and in vivo. J. Exp. Clin. Cancer Res..

[14] Ji Q (2016). Antineoplastic effects and mechanisms of micheliolide in acute myelogenous leukemia stem cells. Oncotarget.

[15] Li J (2018). Natural product micheliolide (MCL) irreversibly activates pyruvate kinase M2 and suppresses leukemia. J. Med. Chem..

[16] Liu Y (2020). Anticancer effects of ACT001 via NF-κB suppression in murine triple-negative breast cancer cell line 4t1. Cancer Manag. Res..

[17] Jin X-H (2018). ACT001 can prevent and reverse tamoxifen resistance in human breast cancer cell lines by inhibiting NF-κB activation. J. Cell Biochem..

[18] Yao S, Ye J, Yin M, Yu R (2020). DMAMCL exerts antitumor effects on hepatocellular carcinoma both in vitro and in vivo. Cancer Lett..

[19] Jaffar J, Glaspole I, Symons K, Westall G (2021). Inhibition of NF-κB by ACT001 reduces fibroblast activity in idiopathic pulmonary fibrosis. Biomed. Pharmacother..

[20] Peng F (2019). Micheliolide ameliorates renal fibrosis by suppressing the Mtdh/BMP/MAPK pathway. Lab. Invest..

[21] Li S (2019). Dimethylaminomicheliolide ameliorates peritoneal fibrosis through the activation of autophagy. J. Mol. Med. (Berl.).

[22] Cao M (2016). The effect of polyene phosphatidyl choline intervention on nonalcoholic steatohepatitis and related mechanism. Am. J. Transl. Res..

[23] Lieber CS (2004). The discovery of the microsomal ethanol oxidizing system and its physiologic and pathologic role. Drug. Metab. Rev..

[24] Maev IV (2020). Effectiveness of phosphatidylcholine as adjunctive therapy in improving liver function tests in patients with non-alcoholic fatty liver disease and metabolic comorbidities: Real-life observational study from Russia. BMJ Open Gastroenterol..

[25] Maev IV (2020). Effectiveness of phosphatidylcholine in alleviating steatosis in patients with non-alcoholic fatty liver disease and cardiometabolic comorbidities (MANPOWER study). BMJ Open Gastroenterol..

[26] Tilg H, Moschen AR (2010). Evolution of inflammation in nonalcoholic fatty liver disease: The multiple parallel hits hypothesis. Hepatology.

[27] Takaki A, Kawai D, Yamamoto K (2013). Multiple hits, including oxidative stress, as pathogenesis and treatment target in non-alcoholic steatohepatitis (NASH). Int. J. Mol. Sci..

[28] Schuppan D, Surabattula R, Wang XY (2018). Determinants of fibrosis progression and regression in NASH. J. Hepatol..

[29] Mridha AR (2017). NLRP3 inflammasome blockade reduces liver inflammation and fibrosis in experimental NASH in mice. J. Hepatol..

[30] Calcagno D (2022). Nlrp3 activation causes spontaneous inflammation and fibrosis that mimics human NASH. Hepatology.

[31] Tsuchida T, Friedman SL (2017). Mechanisms of hepatic stellate cell activation. Nat. Rev. Gastroenterol. Hepatol..

[32] Perakakis N, Stefanakis K, Mantzoros CS (2020). The role of omics in the pathophysiology, diagnosis and treatment of non-alcoholic fatty liver disease. Metabolism.

[33] Xu H (2020). Hepatic proteomic changes and Sirt1/AMPK signaling activation by oxymatrine treatment in rats with non-alcoholic steatosis. Front. Pharmacol..

[34] Xia F (2017). Isobaric tags for relative and absolute quantitation (iTRAQ)-based proteomic analysis of Hugan Qingzhi and its protective properties against free fatty acid-induced L02 hepatocyte injury. Front. Pharmacol..

[35] Yao X (2018). Isobaric tags for relative and absolute quantitation (iTRAQ)-based proteomics for the investigation of the effect of Hugan Qingzhi on non-alcoholic fatty liver disease in rats. J. Ethnopharmacol..

[36] Tacke F, Zimmermann HW (2014). Macrophage heterogeneity in liver injury and fibrosis. J. Hepatol..

[37] Wree A (2014). NLRP3 inflammasome activation is required for fibrosis development in NAFLD. J. Mol. Med. (Berl.).

[38] Leung T-M, Nieto N (2013). CYP2E1 and oxidant stress in alcoholic and non-alcoholic fatty liver disease. J. Hepatol..

[39] Musso G, Gambino R, Cassader M (2009). Recent insights into hepatic lipid metabolism in non-alcoholic fatty liver disease (NAFLD). Prog. Lipid Res..

[40] Sutti S (2014). Adaptive immune responses triggered by oxidative stress contribute to hepatic inflammation in NASH. Hepatology.

[41] Weltman MD, Farrell GC, Hall P, Ingelman-Sundberg M, Liddle C (1998). Hepatic cytochrome P450 2E1 is increased in patients with nonalcoholic steatohepatitis. Hepatology.

[42] Abu-Serie MM, El-Gamal BA, El-Kersh MA, El-Saadani MA (2015). Investigation into the antioxidant role of arginine in the treatment and the protection for intralipid-induced non-alcoholic steatohepatitis. Lipids Health Dis..

[43] Liu Y, Xu W, Zhai T, You J, Chen Y (2019). Silibinin ameliorates hepatic lipid accumulation and oxidative stress in mice with non-alcoholic steatohepatitis by regulating CFLAR-JNK pathway. Acta Pharm. Sin. B.

[44] Gopal T (2020). Nanoformulated SOD1 ameliorates the combined NASH and alcohol-associated liver disease partly via regulating CYP2E1 expression in adipose tissue and liver. Am. J. Physiol. Gastrointest. Liver Physiol..

[45] Seki S (2002). In situ detection of lipid peroxidation and oxidative DNA damage in non-alcoholic fatty liver diseases. J. Hepatol..

[46] Poli G, Biasi F, Leonarduzzi G (2008). 4-Hydroxynonenal-protein adducts: A reliable biomarker of lipid oxidation in liver diseases. Mol. Aspects Med..

[47] Hebbard L, George J (2011). Animal models of nonalcoholic fatty liver disease. Nat. Rev. Gastroenterol. Hepatol..

[48] Bilzer M, Roggel F, Gerbes AL (2006). Role of Kupffer cells in host defense and liver disease. Liver Int..

[49] Jager J, Aparicio-Vergara M, Aouadi M (2016). Liver innate immune cells and insulin resistance: the multiple facets of Kupffer cells. J. Intern. Med..

[50] Miura K, Yang L, van Rooijen N, Ohnishi H, Seki E (2012). Hepatic recruitment of macrophages promotes nonalcoholic steatohepatitis through CCR2. Am. J. Physiol. Gastrointest. Liver Physiol..

[51] Guyot C (2006). Hepatic fibrosis and cirrhosis: the (myo)fibroblastic cell subpopulations involved. Int. J. Biochem. Cell Biol..

[52] Colombatti A, Mucignat MT, Bonaldo P (1995). Secretion and matrix assembly of recombinant type VI collagen. J. Biol. Chem..

[53] Baptissart M (2022). Zac1 and the Imprinted Gene Network program juvenile NAFLD in response to maternal metabolic syndrome. Hepatology.

[54] Oh J (2021). Type VI collagen and its cleavage product, endotrophin, cooperatively regulate the adipogenic and lipolytic capacity of adipocytes. Metabolism.

[55] Herrler M, Bang H, Marahiel MA (1994). Cloning and characterization of ppiB, a Bacillus subtilis gene which encodes a cyclosporin A-sensitive peptidyl-prolyl cis-trans isomerase. Mol. Microbiol..

[56] Zhang H (2017). Elevated serum cyclophilin B levels are associated with the prevalence and severity of metabolic syndrome. Front. Endocrinol. (Lausanne).

[57] Liu L (2021). Giardia duodenalis and its secreted PPIB trigger inflammasome activation and pyroptosis in macrophages through TLR4-induced ROS signaling and A20-mediated NLRP3 deubiquitination. Cells.

[58] Li T (2017). Gastric cancer cell proliferation and survival is enabled by a cyclophilin B/STAT3/miR-520d-5p signaling feedback loop. Cancer Res..

[59] Phillips MC (2013). New insights into the determination of HDL structure by apolipoproteins: Thematic review series: High density lipoprotein structure, function, and metabolism. J. Lipid Res..

[60] Elvira-Torales LI (2020). Consumption of spinach and tomato modifies lipid metabolism, reducing hepatic steatosis in rats. Antioxidants (Basel).

[61] Seth RB, Sun L, Ea C-K, Chen ZJ (2005). Identification and characterization of MAVS, a mitochondrial antiviral signaling protein that activates NF-kappaB and IRF 3. Cell.

[62] Fu J (2022). A conventional immune regulator mitochondrial antiviral signaling protein blocks hepatic steatosis by maintaining mitochondrial homeostasis. Hepatology.

[63] Laizure SC, Herring V, Hu Z, Witbrodt K, Parker RB (2013). The role of human carboxylesterases in drug metabolism: Have we overlooked their importance?. Pharmacotherapy.

[64] Chalhoub G (2021). Carboxylesterase 2 proteins are efficient diglyceride and monoglyceride lipases possibly implicated in metabolic disease. J. Lipid Res..

[65] Santhekadur PK, Kumar DP, Sanyal AJ (2018). Preclinical models of non-alcoholic fatty liver disease. J. Hepatol..

[66] Kohli R, Feldstein AE (2011). NASH animal models: Are we there yet?. J. Hepatol..

[67] Takahashi Y, Fukusato T (2014). Histopathology of nonalcoholic fatty liver disease/nonalcoholic steatohepatitis. World J. Gastroenterol..

[68] Kleiner DE (2005). Design and validation of a histological scoring system for nonalcoholic fatty liver disease. Hepatology.

[69] Ashburner M (2000). Gene ontology: Tool for the unification of biology. the gene ontology consortium. Nat. Genet..

[70] Aleksander SA (2023). The gene ontology knowledgebase in 2023. Genetics.

[71] Thomas PD (2022). PANTHER: Making genome-scale phylogenetics accessible to all. Protein Sci..

[72] Kanehisa M, Goto S (2000). KEGG: kyoto encyclopedia of genes and genomes. Nucleic Acids Res..

[73] Kanehisa M (2019). Toward understanding the origin and evolution of cellular organisms. Protein Sci..

[74] Kanehisa M, Furumichi M, Sato Y, Kawashima M, Ishiguro-Watanabe M (2023). KEGG for taxonomy-based analysis of pathways and genomes. Nucleic Acids Res..

