# Supplementary material for: iTRAQ-based quantitative proteomics analysis of the effect of ACT001 on non-alcoholic steatohepatitis in mice
Source: Sci Rep. 2023 Jul 13;13:11336. doi: 10.1038/s41598-023-38448-4 (PMC10345009; doi:10.1038/s41598-023-38448-4)
Supplement: Supplementary file 1 — Supplementary Information 1. [file 41598_2023_38448_MOESM1_ESM.doc]

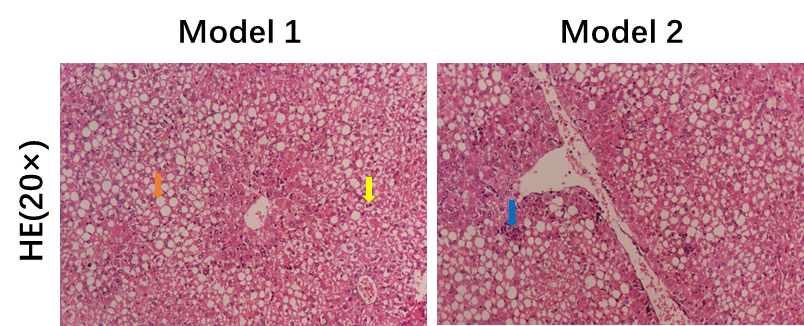


**Supplementary** **Figure S1.** H&E staining results of liver sections from 2 mice in the 2-weekend model group. Yellow arrows indicate hepatocyte swelling; orange arrows indicate hepatocyte steatosis; blue arrows indicate focal necrosis.

**
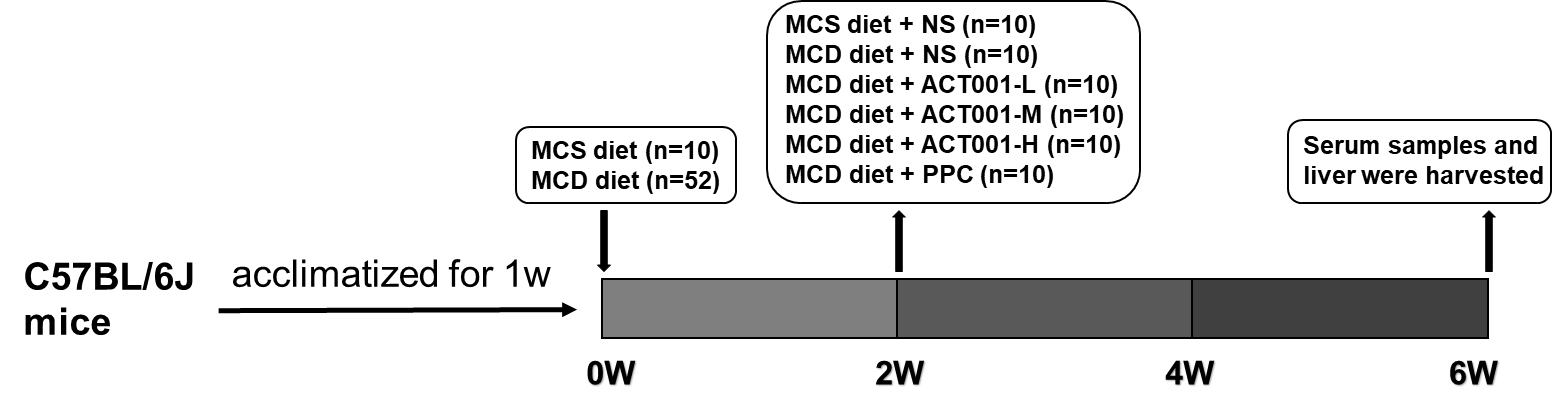
**

**Supplementary** **Figure S2.** Experimental groups and technical route. MCS, Methionine- and choline-sufficient; MCD, methionine-choline deficient; NS, normal saline; ACT001-L, low-dose (100 mg·kg^-1^) ACT001; ACT001-M, medium-dose (200 mg·kg^-1^) ACT001; ACT001-H, high-dose (400 mg·kg^-1^) ACT001; PPC, polyene phosphatidyl choline (150 mg·kg^-1^).

**Supplementary Table S1. Specific primary antibodies used for Western blotting.**

| Antibodies | Company | Product number |
| --- | --- | --- |
| F4/80 | CST | 70076 |
| α-SMA | Abcam | Ab7817 |
| NLRP3 | Abcam | ab263899 |
| CYP2E1 | PROTEINTECH | 19937-1-AP |
| 4-HNE | Abcam | ab243070 |

**Supplementary Table S2. Primer sequences for real-time quantitative PCR.**

| Accession number | Genes | Forward primer (5’-3’) | Reverse primer (5’-3’) |
| --- | --- | --- | --- |
| P06804 | *Tnf-α* | CCCTCACACTCAGATCATCTTCT | GCTACGACGTGGGCTACAG |
| Q02788 | *Col6α2* | CACACAGAATCCAGCTGCCTA | GAACCCGAGGCTTTAGCAGA |
| Q9DCY1 | *Ppib* | TCTCGGAGCGCAATATGAAGG | TACCTTGACTGTGACTTTAGGTCCC |
| Q3V2G1 | *Apoa1* | GCACGTATGGCAGCAAGATG | TTCCTGCAGCTGACTAACGG |
| Q8VCF0 | *Mavs* | CAAATGTTGCCTCTGTTCCCATA | TGATACTGTGACCCCAGACAAGAG |
| Q8QZR3 | *Ces2a* | AGGAGGTACAAGTGTGTCTTCTCAT | TCTTGAAGACCTTGTAGACCACCT |
| P16858 | *Gapdh* | CCTCGTCCCGTAGACAAAATG | TGAGGTCAATGAAGGGGTCGT |

**Supplementary Table S3. The top 20 up-regulated and the top 10 and back 10 down-regulated differential proteins in the MCD group (A) compared to the ACT001-H group (B).**

| **Accession** | **Protein name** | **Gene**  **symbol** | **FC (A vs B)** | ***P* value** | **Adjusted**  ***P* value** |
| --- | --- | --- | --- | --- | --- |
| **Up-regulated protein (Top 20)** | | | | | |
| Q8VG40 | Olfactory receptor 1176 | Olfr1176 | 19.166977690 | 4.37E-03 | 3.15E-02 |
| Q9DCJ9 | N-acetylneuraminate lyase | Npl | 11.727923943 | 3.32E-03 | 2.60E-02 |
| Q8QZR1 | Tyrosine aminotransferase | Tat | 10.576517330 | 7.03E-04 | 9.70E-03 |
| Q3UUI3 | Acyl-coenzyme A thioesterase THEM4 | Them4 | 7.399843706 | 1.70E-03 | 1.71E-02 |
| Q9DBM2 | Peroxisomal bifunctional enzyme | Ehhadh | 6.937207560 | 3.21E-03 | 2.56E-02 |
| A2RS22 | Coronin | Coro1b | 6.742483629 | 2.79E-04 | 5.45E-03 |
| Q9D6E6 | Uncharacterized protein | -- | 6.565632343 | 8.56E-04 | 1.11E-02 |
| P10648 | Glutathione S-transferase | Gsta2 | 6.264598515 | 2.95E-03 | 2.40E-02 |
| Q02788 | Collagen alpha-2(VI) chain | Col6a2 | 5.813087675 | 1.35E-04 | 3.56E-03 |
| Q9D153 | ANK_REP_REGION domain-containing protein | Cdkn2c | 5.509635713 | 3.55E-03 | 2.74E-02 |
| Q9DCY1 | Peptidyl-prolyl cis-trans isomerase | Ppib | 5.427694321 | 9.28E-04 | 1.18E-02 |
| Q9CRB2 | H/ACA ribonucleoprotein complex  subunit 2 | Nhp2 | 5.352059649 | 2.74E-03 | 2.28E-02 |
| Q6QD59 | Vesicle transport protein SEC20 | Bnip1 | 5.269455698 | 9.72E-03 | 4.97E-02 |
| Q99KV1 | DnaJ homolog subfamily B member 11 | Dnajb11 | 5.162154131 | 5.85E-04 | 8.82E-03 |
| Q64460 | Unspecific monooxygenase | 16aoh-b | 5.149407042 | 5.43E-05 | 2.02E-03 |
| Q4VAA9 | Methionine aminopeptidase | Metap1 | 4.955535922 | 8.11E-03 | 4.47E-02 |
| Q3U3K9 | Lipid droplet-regulating VLDL assembly factor AUP1 | Aup1 | 4.421961917 | 5.03E-03 | 3.42E-02 |
| B1AV77 | Aldehyde dehydrogenase | Aldh3a2 | 4.410882367 | 2.86E-03 | 2.36E-02 |
| Q5HZH3 | Apoptosis regulator Bcl-X | Bcl2l1 | 4.387573732 | 7.54E-05 | 2.54E-03 |
| Q3UMW2 | Peptidase_M24 domain-containing protein | Pa2g4 | 4.354550521 | 4.67E-03 | 3.26E-02 |
| **Down-regulated protein (Top 10)** | | | | | |
| Q3TI65 | Mitochondrial 18 kDa protein | Mtfp1 | 0.666282475 | 5.71E-03 | 3.62E-02 |
| Q76MZ3 | Serine/threonine-protein phosphatase 2A 65 kDa regulatory subunit A alpha isoform | Ppp2r1a | 0.664231843 | 6.16E-04 | 9. 19E-03 |
| P19536 | Cytochrome c oxidase subunit 5B, mitochondrial | Cox5b | 0.663545777 | 9.84E-04 | 1.23E-02 |
| Q3V2G1 | Uncharacterized protein | Apoa1 | 0.662995627 | 7.27E-03 | 4.22E-02 |
| Q544R7 | Heme oxygenase | Hmox2 | 0.660884079 | 2.37E-03 | 2.07E-02 |
| Q9D8Y1 | Transmembrane protein 126A | Tmem126a | 0.660693990 | 1.34E-05 | 9.03E-04 |
| Q99KR3 | Endoribonuclease LACTB2 | Lactb2 | 0.659988850 | 9.57E-03 | 4.93E-02 |
| K7N6K9 | Sulfotransferase | Sult2a5 | 0.655245900 | 2.58E-04 | 5.17E-03 |
| Q9D0C4 | tRNA (guanine(37)-N1)-methyltransferase | Trmt5 | 0.654115465 | 1.69E-03 | 1.71E-02 |
| Q9WTP7 | GTP: AMP phosphotransferase AK3, mitochondrial | Ak3 | 0.653201335 | 5.17E-03 | 3.45E-02 |
| **Down-regulated protein (Back 10)** | | | | | |
| Q3TN35 | Uncharacterized protein | Sgta | 0.284436886 | 1.05E-03 | 1.28E-02 |
| P67778 | Prohibitin | Phb | 0.284300398 | 5.95E-05 | 2.15E-03 |
| Q8VCF0 | Mitochondrial antiviral-signaling protein | Mavs | 0.278531740 | 6.91E-05 | 2.38E-03 |
| A0A0A6YVP0 | Immunoglobulin heavy constant gamma 2B | Ighg2b | 0.274187823 | 2.80E-05 | 1.40E-03 |
| Q8QZR3 | Pyrethroid hydrolase Ces2a | Ces2a | 0.272711212 | 4.28E-05 | 1.77E-03 |
| Q9D6J6 | NADH dehydrogenase [ubiquinone] flavoprotein 2, mitochondrial | Ndufv2 | 0.266857856 | 1.25E-06 | 1.91E-04 |
| Q7GIP5 | NADH-ubiquinone oxidoreductase chain 3 | mt-Nd3 | 0.201547792 | 1.11E-08 | 8.03E-06 |
| Q64458 | Cytochrome P450 2C29 | Cyp2c29 | 0.159381803 | 1.63E-05 | 1.00E-03 |
| A4FUW1 | 4932417H02Rik protein | Rptor | 0.132107289 | 5.85E-04 | 8.82E-03 |
| Q9WUD0 | Unspecific monooxygenase | Cyp2b10 | 0.026597645 | 4.99E-12 | 1.44E-08 |

A is MCD group; B is ACT001-H group, n=3 in each group.

[Supplementary Table S4](../Supplementary%20Table%20S4.xlsx)：381 differentially expressed proteins.
